# Interrupting Microaggressions in Health Care Settings: A Guide for Teaching Medical Students

**DOI:** 10.15766/mep_2374-8265.10969

**Published:** 2020-07-31

**Authors:** Rhonda Graves Acholonu, Tiffany E. Cook, Robert O. Roswell, Richard E. Greene

**Affiliations:** 1 Assistant Professor of Pediatrics, Division of Hospital Medicine, Department of Pediatrics, Children's Hospital at Montefiore/Albert Einstein College of Medicine; 2 Interim Director, Office of Diversity Affairs, New York University Robert I. Grossman School of Medicine; 3 Associate Professor of Cardiology and Science Education, Department of Cardiology, Donald and Barbara Zucker School of Medicine at Hofstra/Northwell; 4 Associate Professor of Medicine, Division of General Internal Medicine and Clinical Innovation, Department of Medicine, New York University Langone Medical Center/New York University Robert I. Grossman School of Medicine

**Keywords:** Microaggressions, Bias, Racism, Health Disparities, Empathy, Communication Skills, Cultural Competence, Diversity, Inclusion, Health Equity, Anti-racism

## Abstract

**Introduction:**

Microaggressions are connected to broader conceptualizations of the impact of implicit bias and systems of inequity. The body of evidence supporting the need for more-open discussions in medical education about race, racism, and their impact on health disparities continues to grow. Some have advocated for the importance of bringing anti-racist pedagogy into medical education curricula, which involves explicitly attempting to move beyond people's comfort zones and acknowledging that discomfort can be a catalyst for growth. To discuss the intent and impact of microaggressions in health care settings and how we might go about responding to them, we developed a workshop for third-year undergraduate medical students within a longitudinal undergraduate medical education diversity and inclusion curriculum.

**Methods:**

This workshop occurred during a regularly scheduled clerkship intersession during the 2016–2017 academic year for third-year undergraduate medical students (*N* = 154). Prior to the workshop, the students were asked to anonymously submit critical incident reports on any microaggressions experienced or witnessed to develop case studies for problem-based learning. Teaching modalities included lecture, problem-based learning with case studies, pair and share, and facilitated small- and large-group debriefs.

**Results:**

The session was evaluated using a 4-point Likert scale to assess students' comfort in learning about the information presented. Ninety-eight percent felt confident in identifying microaggressions, and 85% felt confident in interrupting microaggressions when they occur.

**Discussion:**

This personalized workshop exposes students to microaggressions personally experienced by colleagues with an attempt to interrupt them using empathy, awareness, and communication techniques.

## Educational Objectives

By the end of this activity, learners will be able to:

1. Define a microaggression and identify when microaggressions occur in the health care setting.
2. Discuss the importance of power dynamics and intent versus impact as key factors in the overview of microaggressions.
3. Employ strategies to interrupt microaggressions when they occur using a variety of communication techniques.
4. Reflect on the importance of empathy and awareness in understanding the impact of microaggressions.

## Introduction

Racism impacts the health and well-being of patients, from the care we provide to the inequitable outcomes we see in the health disparities literature. Systemic racism also impacts the experiences of students, house staff, and faculty and the climate within which they work and learn together.^1,2^ There is a growing body of evidence to support the need for open and honest discussions about race, racism, and their impact on health disparities in medical education.^3,4^ Recently, there have been calls to action to move beyond these outdated frameworks and to explicitly name and discuss race, racism, and other forms of oppression as a social determinant of health.^1,5,6^ Dr. David Acosta, the AAMC's Chief Diversity and Inclusion Officer, noted that the demographics of the physician workforce impact not only the culture and climate of academic medical centers but also the ways care is provided.^7^ He added that there must be “a deeper focus on changing the culture and climate of our learning and workplace environments,” which requires institutions to be deliberate, intentional, and inclusive.^7^ Existing faculty and student training in medical schools has predominantly focused on cultural sensitivity and implicit bias training, yet this transformative approach to anti-racist and social justice pedagogy requires learners to critically reflect on how power dynamics came to exist and are perpetuated.^8^

Microaggressions, as defined by Derald Sue, are brief, commonplace, daily verbal behavioral or environmental indignities, whether intentional or unintentional, that communicate hostile, derogatory, or negative racial slights and insults toward people of color.^9^ As many microaggressions stem from unconscious, learned ideology, they provide an opportunity to discuss how various forms of oppression can infiltrate our thoughts and behaviors, even when we explicitly value diversity, inclusion, and belonging.

Addressing the impact of oppression and discrimination within medical education curricular content is necessary to recruit and retain a diverse workforce.^5^ The impact of racial bias and structural racism on trainees who are underrepresented in medicine (UIM) is well documented. Differences in educational outcomes for UIM and non-UIM medical students are amplified within medical education, where small differences in assessed performance cascade into larger differences in grades and selections for awards. This amplification cascade can result in long-term consequences for residency and career opportunities. The root causes of this cascade include experiences such as exposure to microaggressions from peers, faculty, and patients, as well as racism.^10–13^ A curricular forum for UIM learners to discuss experiences of discrimination may benefit their well-being. Krieger and Sidney found that “individuals belonging to groups subjected to discrimination may be at lower risk of elevated blood pressure if they are able to articulate, rather than internalize, their experiences of discrimination.”^14^

Although *MedEdPORTAL* has many resources for health professionals to address unconscious bias and racism, as well as guidelines on how to teach cultural competence,^15–17^ the current curriculum is unique in that it directly defines and addresses microaggressions. Similar to other published work, it uses cases gleaned from experiences of trainees, which provide for robust discussion.^18^ Yet it offers a distinct approach to focus on microaggressions in small- and large-group formats.

## Methods

At the NYU School of Medicine, the Office of Diversity Affairs and Office of Medical Education collaborated to develop and implement curricula focused on diversity and inclusion, including content focused on race, gender, sexuality, and identity across the undergraduate medical education continuum. This workshop, which was placed in the fall interclerkship, was utilized to introduce the concept of microaggressions, their impact, and ways to address them in the clinical setting.

This curricular intervention occurred during a regularly scheduled clerkship intersession during the 2016–2017 academic year for third-year undergraduate medical students (*N* = 154). The demographics of this class, identified from admissions data, included 53% males and 46% females, with 14.6% of the students self-identified as UIM, 36.7% as Asian, and 53.2% as White. This total was over 100% as some students self-identified as belonging to two racial/ethnic categories. Prior to the workshop, the entire student body was asked to anonymously submit critical incident reports on any microaggressions they had experienced or witnessed to develop case studies for problem-based learning. Two facilitators—one faculty member and one diversity affairs administrator—were paired to guide the 90-minute workshop. The ratio of students to facilitators was 20:2. Teaching modalities included lecture, problem-based learning with case studies, pair and share, and facilitated small- and large-group debriefs.

This resource was developed by a team of health professionals within the Office of Diversity Affairs at NYU. Designed to be used as a stand-alone session or as part of a larger curriculum addressing race, gender, sexuality, and identity, it had no formal prerequisites. The session was targeted to students in their clerkship year and timed to occur during a regularly scheduled interclerkship intensive (ICI) week when all students returned to campus for classroom-based activities. We selected this timing in the overall curricular structure to take advantage of students' early experiences in patient care and in working within the hierarchical organizational structure of discipline-specific teams. We conducted the 90-minute interactive lecture and small-group session for 154 students with eight instructors. We chose to run this workshop during ICI week, but it could be run at any time when learners convene in a medical educational curriculum.

### Preworkshop Survey

A Google form was created to serve as the pre-ICI survey (Appendix A). Four weeks prior to the workshop, the survey was sent via email to all clerkship students. They were informed that a workshop on microaggressions would be occurring during their upcoming ICI week and that it would be helpful to have their own experiences included. The idea was to use critical incident reporting^19^ in order to effectively enhance the conversation. Additionally, these examples would provide a lens through which students who were previously unfamiliar with or unaware of microaggressions could gain insight into the experiences of their peers, particularly those holding identities different from their own. The survey asked students to anonymously submit examples of anything they had experienced that could be classified as a microaggression. They were then asked to identify how they would label the microaggression (e.g., racist, sexist, ableist, heterosexist, classist, cissexist, xenophobic, etc.).

The survey was closed 48 hours prior to the first workshop. The team reviewed the submissions, selecting and deidentifying examples for use in the various sections of the workshop.

### Facilitator Development

All facilitators were selected based on interest in the topic and prior participation in discussions with the Office of Diversity Affairs about education and experiences related to power dynamics within medicine. Faculty willingness to examine discomfort as it related to race, power, and privilege was a helpful attribute in selecting facilitators for this workshop. Eight facilitators were chosen to participate. All eight self-identified as racial and/or sexual and gender minority faculty or staff members. Although these demographics were not proportional to those of the students, faculty who held a minoritized identity noted the personal relevance of the topic. *MedEdPORTAL* has published curricula that could be used to assist faculty in discussing identity and privilege as preparation for this type of workshop.^20^ Since staff in the Office of Diversity Affairs had facilitated discussions about explicit and implicit discrimination in medicine, we paired Office of Diversity Affairs staff with other facilitators. Once facilitators had been confirmed, the Office of Diversity Affairs hosted hour-long faculty development sessions 1 day prior to the workshop.

During the faculty development session (Appendix D), the facilitators' guide was reviewed extensively. The facilitator's guide (Appendix B) featured a detailed breakdown of the flow of each section of the workshop, including suggested prompts and ideas. The guide also listed the overall objectives of the workshop, along with key concepts to be emphasized during each section and resources invaluable for discussing microaggressions. Also during the faculty development session, the foundational principles of microaggressions, power, and oppression were described. There was a practice session of the workshop led by the Office of Diversity Affairs facilitators. We reviewed possible responses to the cases and also addressed questions that were likely to arise. Additional opportunities to review possible planning for any foreseeable challenges were also discussed.

### Workshop

Sessions were scheduled for 90 minutes, and students were divided into groups of 20 alphabetically with one facilitator.

#### Introductions/group agreements

The workshop began with a brief introduction of the facilitator as well as their role within the Office of Diversity Affairs and their interest in this content/topic. After the agenda for the workshop had been reviewed, we used poster paper and markers to develop group agreements on the communication approaches and expectations during the workshop. The purpose of the group agreements was to actively engage the students in creating shared rules about how communication would be handled in the workshop. Ideas such as not sharing information heard in the session without the permission of the student and allowing students an opportunity to be heard if they wanted to talk were frequently cited as important for group agreements.

#### Large-group discussion

After the group agreements had been discussed and agreed upon, we moved into a large-group discussion with all 20 students in the room, using a slide presentation as a guide (Appendix C). We discussed the impact of communication on outcomes and reviewed the basic definition of microaggressions. We then discussed possible ways to address microaggressions. Although students and facilitators could be left to come up with possible ways to address microaggressions when they occur, outside resources could also be used to help guide the discussion, such as the previously published Interrupting Microaggressions tool (Appendix B, pp. 17–18).^21^ In addition, using previously learned materials from a preclerkship doctoring course could provide a bridge between the curricula learned in various courses. The acronym PEARLS was used to discuss building relationships with patients in our doctoring course; the PEARLS components of partnership, empathy, apology, respect, legitimation, and support could be applied when discussing how to approach microaggressions.^22^

#### Small-group discussion

Each group of 20 students was then split into four small groups of five students each. The groups reviewed and discussed the previously collected examples of microaggressions. Students were instructed within their small groups to discuss the categorization of the microaggression (e.g., racist, sexist, ableist, heterosexist, classist, cissexist, xenophobic, etc.) and the power dynamics within the cases. They then answered questions about strategies they would have employed to respond to the microaggression presented. The students next chose a speaker for their group and reported out the highlights of their discussion to the larger group. Themes that typically emerged during these discussions were the following:

• The impact of exposure to microaggressions on both the recipient and others present, including patients;
• Student techniques in supporting peers who were subject to microaggressions; and
• Being cautious about responding to a microaggression for another person and usurping their power.

#### Pair role-play

For the remainder of the session, students were paired up. Using the Interrupting Microaggressions tool (Appendix B, pp. 17–18), they were asked to review various statements and discuss their harm and any responses to them.

#### Reflection

For the final 15 minutes, the groups were asked to reflect, first quietly on their own and then as a large group, on the applicability of the tools used in the session and on how likely they were to use them as they continued in the clerkships.

#### Wrap-up

At the end of the session, we highlighted mistreatment-reporting mechanisms and reminded learners of student health services, including identity-focused mental health services, such as our affinity group (people of color, LGBTQ+, etc.) therapy sessions.

### Evaluation

After the session, the students were emailed an evaluation (Appendix E) to assess their overall impression of the session as well as share any positive or negative comments. Facilitators participated in a postworkshop debriefing that centered on three questions (Appendix F): What went well? What could be improved? What challenges/pitfalls did you face?

## Results

This curriculum was first implemented for 154 second-year students in their clerkship years during the 2016–2017 academic year. Overall, the session was well received by students and faculty. This project met the NYU School of Medicine's criteria for certification as a quality improvement project, not a human subjects research project, based on a self-certification process.

### Results of Student Satisfaction Surveys

Students were asked to complete an evaluation distributed online by the Office of Medical Education. Both quantitative and qualitative data were obtained. A total of 82 students responded to the postworkshop survey, giving us a response rate of 53%. Of those who responded, 98% felt confident in identifying microaggressions at the end of the workshop, 85% felt confident in interrupting microaggressions when they occur, and 99% felt confident in supporting their peers and colleagues when they experience microaggressions (Table 1).

**Table 1.**
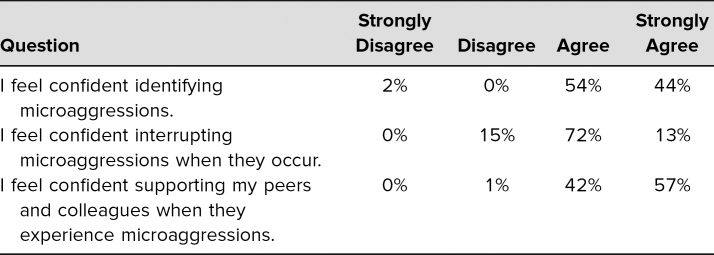
Student Evaluations of Workshop (*N* = 82)

Evaluations revealed that 100% of the students thought the facilitators were well prepared. Ninety-eight percent indicated that the facilitators created a welcoming and inclusive environment for discussion, and 99% felt that the facilitators effectively communicated the session information (Table 2).

**Table 2.**
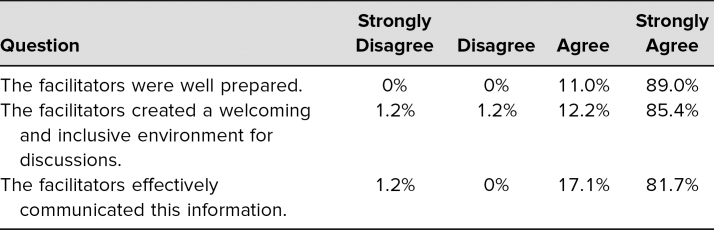
Student Evaluations of Facilitators (*N* = 82)

Narrative comments provided by students were primarily organized around three themes: the environment, the impact of the facilitators on that environment, and the cases themselves. In terms of the learning environment, students commented positively on the space that was created in order to have an open discussion:

• “I enjoyed the open and accepting atmosphere of the workshop. It was important that it was clearly specified that the goal was to raise awareness and become allies.”
• “I liked that this was an open conversation where we were really free to share our opinions. There were no feelings of judgment.”

With respect to the facilitators' impact, students commented as follows:

• “Dr. X is a very engaging group leader who managed to create a space where many people, who had previously stated they would not be able to/did not want to discuss microaggressions, began talking and bringing up their own experiences.”
• “I liked that the preceptors shared their experiences.”

Examples of cases submitted in the responses included the following:

• “As a female, someone shouted, ‘hey nurse’ as I walked down the street with a male medical student. We were both wearing a white coat and scrubs.”
• “At the free clinic, a student was using the wrong pronouns for a transgender patient.”
• “I went to pick up my scrubs and had my tattoos visible. All the doctors ignored me when I asked for assistance. When I went back later with my tattoos covered, everyone was much friendlier.”

Students also noted the importance of reviewing real cases that they and/or their colleagues had experiences:

• “I enjoyed reviewing REAL submitted cases. It demonstrated that these microaggressions really do happen.”
• “Sharing our experiences was very valuable.”

### Reflections From Facilitators

Following the workshop, the facilitators were emailed a list of reflection questions (Appendix F). The facilitators then met to discuss their impressions of the workshop. All found the workshop to be very valuable for both the students and themselves. Facilitators stressed the importance not only of creating group agreements but of asking each student to commit to observing the parameters discussed. In addition, facilitators noted the importance of stressing the difference between the intention and the impact of microaggressions. As microaggressions are not usually said with an intent to be offensive, it could sometimes be difficult for some to understand the negative impact. The facilitators noted the importance of providing consistent messaging that the impact can have unintended consequences. Those unintended consequences, moreover, might be experienced by only a single individual, yet that would not negate the importance of the interaction.

Another major area noted by the facilitators was the importance of providing concrete quick tools that could be used in the moment by students to interrupt microaggressions when they occur. The tools (Appendix B) could also be used by third-party witnesses to an interaction, which gave the students dual opportunities to offer support and encouragement to their peers.

## Discussion

We successfully developed and implemented a feasible and efficient curriculum to introduce clerkship students to the concept of microaggressions, as well as specific tools to try to address them in a way that would be meaningful and effective in a variety of settings. The overall objectives of the workshop, which included defining microaggressions and using communication techniques to increase awareness and provide an opportunity to interrupt microaggressions, were met.

Utilizing real cases that students had personally experienced provided a level of personal connection they appreciated. It also gave legitimacy and validation to the experiences of their peers. Offering students an opportunity to openly share their ideas and their understanding, or lack thereof, in an environment that felt safe for discussion proved to be invaluable to the participants. Given the hierarchy and apprenticeship model in medicine, power dynamics are associated with many of the microaggressions presented in this workshop. As the focus of this workshop was on the students, the communication strategies discussed were primarily utilized to empower them with tools in the moment.

This resource adds to the growing body of literature on medical education curricula focused on diversity and inclusion content. Because health disparities continue to be a significant contributor to differences in care, the hope is that this type of resource will have a positive impact on learners, learning climate, and, most importantly, patient care. One way to do that is to improve communication skills of medical students in how to approach difficult or uncomfortable conversations regarding race, gender, and other topics. In addition, teaching students about empathy and how to support each other is crucial to fostering a generation of physicians who, we hope, will put an end to the communication gaps that contribute to health disparities. Learners valued the opportunity to increase their awareness and become allies with their colleagues in an effort to proactively eliminate entrenched stereotypes and biases.

It is critically important to support all learners in their identity development processes. Learners hold a multitude of identities that impact their experiences in medicine. While our workshop addresses the discrimination of many minoritized groups, conversations about racism, diversity, and inclusion can be difficult to facilitate without proper training and preparation due to the powerful emotional responses to race-related content, as Tatum has noted.^23^ While there has been little research on how medical students develop racial identity, the broader life span development literature provides a framework for racial identity development that can be applied.

A limitation of this curriculum is the paucity of published data in the medical education literature establishing a link between the importance of discussions about microaggressions and any effective outcomes for patients, students, and/or the learning environment. Studies in the social science and psychology literature suggest the importance of these conversations but have not been cited as widely in the mainstream medical education literature.^24–26^ We did query the students about their plans to change behavior, yet there are no follow-up outcome data to identify if any changes in behavior have actually occurred. Future iterations of this curriculum should include interval follow-up assessments of students to identify sustainability. Another limitation, the use of a 4-point Likert scale without a neutral option, was a deliberate approach to require the trainees to declare an opinion. A core component of this workshop is the concept of easing trainees out of their comfort zone, which led to the decision to also require an opinion on the evaluations. Implications of such an approach include the possibility of a decreased response rate given the lack of a neutral response, which would impact the results. A 5-point Likert scale could easily be substituted in order to allow for a neutral response.

As noted by Acosta and Ackerman-Barger,^3^ facilitating content of this nature can be challenging for faculty members unfamiliar with microaggressions. Faculty members must have a depth of knowledge about power dynamics when discussing these microaggressions or incorrect messages can be delivered to unknowing learners. For example, when discussing the microaggression of female doctors being assumed to be nurses, it is important to note that the microaggression is not about a hierarchical relationship between doctors and nurses, as both professions are rigorous, essential, and honorable. Instead, it is about the power dynamic involved in being assumed to hold a role different than one's actual role simply because of one's identity. Similarly, in the microaggression about a Black student being asked if they are “transport,” there are scenarios in which the patient may have been waiting a long time for a study or had recently been told they would be transported that may, from the patient's perspective, be less about the student's race than other scenarios. However, it is important for the facilitator to note that regardless of the intention, the student is still left with the fear, anger, or embarrassment of wondering whether their race was at the foundation of this question.

Conversations such as this can be overwhelmingly stressful for faculty. Therefore, it may be difficult to find facilitators who are willing or have the knowledge to engage in these conversations. Having said that, many students noted anecdotally that they appreciated the opportunity to have these discussions and for someone to acknowledge that these situations were occurring. For those identified groups that experience microaggressions more frequently, it was validating to begin the conversation.

For the faculty members who do facilitate these workshops, we recommend cofacilitating in pairs, to have support when unexpected challenges arise. Having a cofacilitator can lessen the emotional burden for each individual facilitator. Furthermore, debriefing the sessions not only can be helpful for refining future iterations but also can provide much-needed emotional support to faculty, many of whom may hold marginalized identities. Teaching about microaggressions requires at least an intermediate level of subject matter expertise in diversity, equity, and inclusion. Schools seeking to include this content should be mindful of the minority tax and avoid tasking underrepresented faculty with providing this training. Therefore, we support training any faculty member interested in diversity, equity, and inclusion efforts to teach this content, but we do not feel it is appropriate or advantageous to mandate or require all faculty to do so, given the sensitive nature of the topic.

A future direction for this curriculum would be to repeat it on an annual basis and to encourage periodic submissions of microaggressions in an anonymous fashion as they relate to the clinical learning environment. In addition, following up with students to identify opportunities to interrupt microaggressions would aid in understanding if the curriculum has sustainable outcomes. These conversations occur best in small groups, and we hope that these repeated discussions about exposure to microaggressions and discrimination can be integrated into a longitudinal curriculum in the clerkship years. Training additional clerkship faculty facilitators in the definition of microaggressions and communication strategies to interrupt them may encourage a more-open dialogue as students rotate through the various clerkships. Additional support from faculty who actively interrupt microaggressions will provide modeling for students who may not always feel empowered or have the agency to address microaggressions given the power dynamics and hierarchy within medicine. Success of this curriculum will be demonstrated by sustainable integration of these conversations into the clerkships accompanied by demonstrable positive outcomes of effective change to the learning environment and improved patient care.

## Appendices

Preworkshop Survey.docxFacilitator Guide.docxWorkshop Presentation.pptxFaculty Development Agenda.docxPostworkshop Evaluation Form - Students.docxPostworkshop Debriefing Questions - Faculty.docx All appendices are peer reviewed as integral parts of the Original Publication.
