# Supplementary material for: Interrupting Microaggressions in Health Care Settings: A Guide for Teaching Medical Students
Source: MedEdPORTAL. 2020 Jul 31;16:10969. doi: 10.15766/mep_2374-8265.10969 (PMC7394346; doi:10.15766/mep_2374-8265.10969)
Supplement: Supplementary file 1 — Preworkshop Survey.docxFacilitator Guide.docxWorkshop Presentation.pptxFaculty Development Agenda.docxPostworkshop Evaluation Form - Students.docxPostworkshop Debriefing Questions - Faculty.docx [file mep_2374-8265.10969-s001.zip › A. Preworkshop Survey.docx]

**Interrupting Microaggressions in Health Care Settings: A Guide for Teaching Medical Students**

Pre-Workshop Survey

Microaggressions can be defined as brief and commonplace daily verbal, behavioral, or environmental indignities, whether intentional or unintentional, that communicate derogatory, hostile or negative slights and insults toward people of marginalized experiences (i.e. race, sexual orientation, class, etc.)

If you feel like you have experienced a microaggression during your time as a medical student, please describe it here.

How would you label this microaggression?

- Racist
- Sexist
- Ableist
- Heterosexist
- Classist
- Cissexist
- Xenophobic
- Other - ______________________________

Thank you for sharing your experiences with us. If you would like to submit multiple responses, you are welcome to do so.
