# Supplementary material for: Interrupting Microaggressions in Health Care Settings: A Guide for Teaching Medical Students
Source: MedEdPORTAL. 2020 Jul 31;16:10969. doi: 10.15766/mep_2374-8265.10969 (PMC7394346; doi:10.15766/mep_2374-8265.10969)
Supplement: Supplementary file 1 — Preworkshop Survey.docxFacilitator Guide.docxWorkshop Presentation.pptxFaculty Development Agenda.docxPostworkshop Evaluation Form - Students.docxPostworkshop Debriefing Questions - Faculty.docx [file mep_2374-8265.10969-s001.zip › B. Facilitator Guide.docx]

**Interrupting Microaggressions in Health Care Settings: A Guide for Teaching Medical Students**

**Facilitator Guide**

**Format**: Workshop

**Timeline**: 1.5 hours

**Objectives**:

At the end of this curricular intervention, participants will be able to:

1. Define a microaggression and identify when microaggressions occur in the health care setting.
2. Discuss the importance of power dynamics and intent vs. impact as key factors in the overview of microaggressions.
3. Employ strategies to interrupt microaggressions when they occur using a variety of communication techniques.
4. Reflect on the importance of empathy and awareness in understanding the impact of microaggressions.

**A/V Needs**: Screen and powerpoint presentation

**Powerpoint**: yes – Appendix C

**Handouts**:

1. Infographic: Intent and Impact
2. Infographic: Interrupting Microaggressions
3. Tool: Interrupting Microaggressions (handout with columns)
4. Microaggression Cases (each case on its own individual slip of paper)
5. Pairs Activity: Microaggressive Statements

**Other materials** **needed**: Poster paper, markers

**LESSON PLAN**

***Introduce Faculty and Framing the Workshop (2 minutes)*** *– Slide 1*

- Introduce self and any additional faculty facilitators. Comment on interest in facilitating this workshop. Acknowledge that this topic is difficult and has the potential to make people uncomfortable. Acknowledge that the diversity of opinion and the opportunity to explore views and assumptions are what will make the workshop successful. State that each person brings a unique perspective to this conversation, varying levels of experience with the topic, and that no one is perfect. Generally no one comes to medical school, as student or faculty, intending to hurt or offend other people. The only way we can learn is to take some risks and to engage in a dialogue around difficult topics like this. Remind them that the examples used in class today were taken from their classmates, anonymously, and so they need to be especially mindful and respectful when discussing them.

***Agenda (1 minute)*** *– Slide 2*

- Briefly review the agenda.

***Develop Group Agreements (7 minutes)*** *– Slide 3*

- Use poster paper and markers to develop group agreements together
- Ask students to add to the list.

Suggestions for additional group agreements:

- - “Vegas” rule – “What happens in Vegas, stays in Vegas.” Please don’t tell any stories fellow students share outside of this room without their explicit permission.”
  - “Make space, take space” - If you are generally a more talkative person, please allow others to speak. If you generally don’t speak up, you are encouraged to do so here.
  - “One mic” – only one person should be speaking at a time.
  - Take care of yourself - Some of these microaggressions may be triggering for some students. Please do what you need to do to take care of yourself throughout this session, including excusing yourself to take some time or space if needed. Remind students of the opportunities to report mistreatment when it occurs as well as the services available including the various student affairs offices, offices related to diversity, equity and inclusion as well as offices for counseling and therapy.
  - Listen. Encourage the students to listen to each other and dialogue with each other as well as with you as the facilitator.

***Group Discussion (20 minutes)***

*This first part of the discussion is about understanding the difference between intention and impact.*

- How does our communication affect our outcomes? – Slide 4
- Review the “Intent and Impact” infographic (Pass out the handout to all students) – Slide 5
- Brainstorm with the group – Have you ever experienced unintended consequences when you were trying to communicate with someone/about something?

This next part of the discussion is understanding the definition of a microaggression.

- Brainstorm with group: Create a working definition of a microaggression.
- Review one possible definition of a microaggression – Slide 7
- Microaggressions can be separated into 3 categories as defined by Sue et al.
  - Microassaults – explicit derogations meant to hurt the intended victim.
  - Microinsults – behavioral/verbal remarks that convey rudeness, insensitivity and demean a person’s heritage and/or identity.
  - Microinvalidations – verbal comments or behaviors that exclude, negate or nullify the psychological thoughts, feelings or experiential reality of a person.
- Provide an example of a sexist microaggression – Slides 8-9
  - Brainstorm with the group – what is harmful about this microaggression? – Slide 10
  - *The assumption is that it is okay to refer to a female student in a non-professional manner where that would not be done to a male student.*
  - *Consider exploring the implicit bias that exists where women may not be seen as the physician and that comments such as may perpetuate that belief.*
- Review all the “isms” that a microaggression can be – Slide 11
  - Note that there are many more “isms” that may be microaggressions. Dr. Sue’s original work focuses on racial microaggressions. Highlight that his future work notes the inclusion of any marginalized person can experience microaggressions.
  - Again, note that often times microaggressions are not the intended outcome, but are a result of being unaware of the consequences of our communication.

This next part of the discussion focuses on what to do when you experience/witness a microaggression.

- So you witness or experience a microaggression – what can you do about it? Review the interrupting microaggressions infographic and the tool for interrupting microaggressions – slides 12-16. Pass out the “Interrupting Microaggressions” infographic.
  - Slide 12: “Ack!You just witnessed a microaggression.” Acknowledge that in the moment, you may have a range of emotions. Explore those emotions. Discuss that they may range from anger to surprise to frustration to agreement.
  - Slide 13: “In the moment you can…” Acknolwedge that you can do several things in the moment when a microaggression occurs.
  - Slide 14: “Separate the person from the behavior. For example, “that could be perceived as a racist remark,” rather than “You’re racist.” It is important to the importance of intention vs. impact and highlight here that the person who stated the comment may have not have intended for those comments to be perceived that way.
  - Slide 15: “Ask questions about the behavior using “How…” or “What made you” rather than “why” as this can make folks defensive.” Again, acknowledge the role of intention vs. impact in microaggressions and use language that seeks to explore the assumption or stereotype that is at the root of the microaggression.
  - Slide 16: Use “I” statements describing how the action affected you or refer to the action indirectly. For example, “When ____ happened, I felt _______.” Acknowledge the importance of using “I” statements when responding to microaggressions so that you are owning your response. For example, you should not lead with a statement such as, “Do you know how that must have made that person feel?” as you are not able to know that for sure.
  - Slide 17: Note the tone of voice, body language, etc. when responding. What is your intention and goal for your outcome? How can your tone and body language help you achieve this?” Acknowledge the role of non-verbal behaviors in the response to a microaggression. Again, using the intention vs. impact model allows someone to explore the rationale behind the microaggression. Therefore, using tone of voice and body language that is aligned with exploration as opposed to assumptions may aid in the process. In addition, given the power dynamics within medicine, the recipient of the microaggression and/or the person who witnessed the microaggression may not feel that they are in a position to address the “deliverer” of the microaggression. In those situations, one could consider the role of body language in offering gestures of support in the moment.
- How can you offer support to somebody you just witnessed experience a microaggression? – Slide 18. Brainstorm ideas with the group. Discuss these ideas both from the perspective of someone who have may have said something to the person who delivered the microaggression as well as if the person witnessed the microaggression but did not say anything. Explore feelings of helplessness, lack of power, agreement and feelings of being unsure of what to do.
- PEARLS was learned back in doctoring courses (referred to as the “Practice of Medicine”) but has variable names in other institutions. Recall this acronym which was used as a way to recall how to build relationships with patients and can be used in situtations to help your colleagues when they experience a microaggression. – Slide 19
  - P=Partnership. The function is for joint problem solving.
  - E=Empathy. The function is to show understanding and compassion about illness.
  - A=Apology. The function is to show concern for errors and hurts. Say “I’m sorry” for hurting/offending you.
  - R=Respect. The function is to value the person’s choices, traits, behaviors and special qualities.
  - L=Legitimation. The function is to let the person know that their response is normal and respected. Validate their response.
  - S=Support. The function is to let the person know that you are not abandoning them and you will be there to help them.

***Small Groups – Microaggression Cases - (30 minutes)***

- Separate the students into small groups of 4-5 student each. Provide the students with markers and poster paper.
- Pass out the “Tool: Interrupting Microaggressions.”
- Have each group pick a microaggression case from the handout. Remind the students that these are cases submitted by their colleagues so to please be respectful of how they are discussed.
- Give the group these instructions:
  - Read the case aloud (5 minutes)
  - Identify how the case is an example of a microaggression; specifically, identify the marginalized group and the assumption made or bias toward that group.
  - Imagine that one of your peers is relaying this story to you. Identify an empathic statement that you could make to your peer after hearing this story.
  - Discuss the pros/cons of “interrupting” the microaggression, or “speaking up” in the moment. Remember microaggressions do not need to be reflexively handled. The first step is deciding whether to and when to interrupt a microaggression. Discussing microaggressions with colleagues or friends are completely valid ways of dealing with microaggressions in the hospital setting.
  - When deciding whether or not to speak up, consider:
    - the power dynamic between the person who is the “recipient” of the microaggression and the “deliverer”- how would changing this power dynamic make it easier to speak up?
    - the nature of the relationship (whether there was a prior relationship, whether it is or will be ongoing/longitudinal or whether it is a one time encounter)
    - the potential repercussions for the person speaking up, the “deliverer,” the relationship, the community at large
    - the setting and timing- what is the right place and the right time?
    - whether or not the “deliverer” is a member of the marginalized or targeted group
  - Thave the group answer the following questions about their selected case and write their responses on the poster paper (10 minutes):
    - Why is this microaggression inappropriate?
    - How would you respond to this microaggression? Encourage the group to use the Tool:Interrupting Microaggressions for ideas for responses.
    - What strategies would you emply in your response?
  - Have the students report back to the large group, sharing the case as well as the main ideas from their responses (15 minutes).

***Pair Role-play: Microaggressive Statements (15 minutes)***

- Provide all students the “Pairs: Microaggressive Statements” sheets.
- Ask students to pair up and determine why these statements could be considered microaggressive. Specifically ask them to identify (1) what marginalized group is being targeted and (2) what assumptions are being made or what bias is being expressed toward that group.

***Conclusion – Reflection (15 minutes)***

- What are your initial responses to this activity?
- How do you plan to use these tools moving forward?
- How do you plan on being accountable to each other in ensuring that this is sustainable?
- Please complete the evaluation (Appendix E). Consider making the evaluation with a platform that can be done on their mobile devices to aid in the efficiency of completing it.

**Small Group Activity: Microaggression Cases**

Instructions: Separate this into strips of paper with one case each. Hand out one to each small group.

**Case 1a: Supervisor to you**

I’m really excited to put together my ERAS application for a surgical residency. When I go to talk to my resident, who happens to be a man, about my career plans he says, “As a woman, you should really think about your ability to have a family before going into surgery.” I know from previous conversations that he has three kids at home. I point out that he was able to become a successful surgeon and has a family, but he continues to not take me seriously and brushes me off.

**Case 1b: Supervisor to you**

During one of my rotations, I am paired with another medical student who happens to be white. I am not. As we work with the attending, it becomes clear through eye contact, or lack thereof, that the attending doesn’t seem to like me. As the attending and the white medical student continue to become well-­‐acquainted throughout the day, the attending at one point says to them, "You know, you remind me of my daughter."

**Case 2: Supervisor to patient**

I am working with a gay man who just decided to start pre-­‐exposure prophylaxis (PrEP) and one of my attendings comments that he thinks this decision is very responsible of the patient. I agree that this is a great preventative measure. I am shocked when he then comments that he finds the patient’s behavior, having receptive anal sex, irresponsible and that he thinks it’s morally wrong to be gay.

**Case 3a: Supervisor to another person on your team**

We’re on a break from seeing patients and my attending starts talking about all the LGBT people in one of the classes, identifying and “outing” them without their permission. I have some queer and trans friends and know this is not only disrespectful, but can be dangerous. I feel really uncomfortable that this happened.

**Case 3b: Supervisor to another person on your team**

During my clerkship rotations, one of my team members is constantly confused with another Asian female medical student. It really upsets me when I hear attendings calling her by our other classmate’s name and asking the residents "which one is which?"

**Case 4a: Peer to peer**

I went to an Ivy League college for undergrad, had a 4.0 GPA, and am first-­‐author on a publication in a high-­‐impact medical journal. Yet my fellow students seem to believe I was only accepted to NYU School of Medicine because I’m black, not because of my academic achievements. This has left me feeling depressed and like my achievements aren’t valid.

**Case 4b: Peer to peer**

I’m working on a group project and I’m the only woman in the group. Every time we divide tasks, I’m asked to do all the secretarial type work (scheduling, emailing, printing, calling) for the meetings, but the men never assign themselves these kinds of tasks. I can’t help but wonder if they think I’m less capable than they are.

**Case 5: Patient to you**

I’m a black woman and when I go to see a white patient during rounds, she starts screaming at me, “Why are you here?!” As I gape at her and try to come up with a response, she says, “Why would they give YOU my medical information?"

**Case 6: Patient to someone else on your team**

I never really thought about male privilege until I was on the wards. When I walk around with my women colleagues, patients always assume they are nurses, even though they are wearing white coats. That never happens to me.

**Case 7: Faculty to faculty**

I am a part of an advisory board where I am the only person of color, everybody else is white. We have been tasked with advising the hospital on all things related to the patient experience. This board will likely shape some policy and even the aesthetics of the hospital. I mentioned that the board does not represent the diverse population we serve and that we should invite more diverse folks to our future meetings. It’s been 6 months and nothing has changed.

**Small Group Activity: Microaggression Cases with Responses**

**Case 1a: Supervisor to you**

I’m really excited to put together my ERAS application for a surgical residency. When I go to talk to my resident, who happens to be a man, about my career plans he says, “As a woman, you should really think about your ability to have a family before going into surgery.” I know from previous conversations that he has three kids at home. I point out that he was able to become a successful surgeon and has a family, but he continues to not take me seriously and brushes me off.

- Why is this microaggression inappropriate? *This microaggressions makes assumptions that the person wants a family and has its roots in the stereotype that women are primarily responsible for family planning. The power dynamic may make this microaggression difficult to challenge as the person is asking their resident for advice. It is unclear if this resident may also have a role in this person’s evaluation which can make it more challenging.*
- How would you respond to this microaggression? *Multiple strategies could be used to address this microaggression. If the responder wants to address the assumption that she wants a family, she could say, “I am wondering if you are assuming that because I am a woman, I want to have a family.” Or a more direct comment could be, “that could be interpreted as a sexist remark.”*
- What strategies would you employ in your response? *When attempting to interrupt this microaggression, paying attention to tone and body language is critically important. It is also important to use “I” statements and to separate the comment from the behavior. For example, it would be important to not say, “that’s so sexist.”*

**Case 1b: Supervisor to you**

During one of my rotations, I am paired with another medical student who happens to be white. I am not. As we work with the attending, it becomes clear through eye contact, or lack thereof, that the attending doesn’t seem to like me. As the attending and the white medical student continue to become well-­ acquainted throughout the day, the attending at one point says to them, "You know, you remind me of my daughter."

- Why is this microaggression inappropriate? *This microaggression is challenging because the deliverer likely does not see the effect that this could be having on those around them, therefore highlighting a critical role of intention vs. impact. The student feels that the attending shares more in common with the other student and therefore, is struggling to see how their worth can be validated when they are not ascribed to the same racial identity as the other student. Again, power dynamics are definitely at play in this microaggression as the attending is the supervisor for the students.*
- How would you respond to this microaggression? *Many assumptions could be at play in this microaggression. It would be important to separate the comment from the overall goals of the rotation, acknowledging the assumption that a student who reminds the attending of their offspring may be more likely to earn a better grade or receive preferential treatment. Therefore, an attempt can be made to restate the comment and explore its meaning – “I heard you say that X reminds you of their daughter and I am wondering if those traits are being used in the overall evaluation process.”*
- What strategies would you employ in your response? *Acknowledging the power dynamics as well as understanding the role of intention vs. impact would be important strategies to employ. It would be an important discussion to highlight when certain microaggressions might be better addressed by faculty at similar levels so a response could include meeting with the clerkship director to address this in a more formal way.*

**Case 2: Supervisor to patient**

I am working with a gay man who just decided to start pre-­‐exposure prophylaxis (PrEP) and one of my attendings comments that he thinks this decision is very responsible of the patient. I agree that this is a great preventative measure. I am shocked when he then comments that he finds the patient’s behavior, having receptive anal sex, irresponsible and that he thinks it’s morally wrong to be gay.

- Why is this microaggression inappropriate? *This microaggression has its roots in homophobia. The deliverer of the microaggression is casting judgment on a patient. The power dynamic and hierarchy can be particularly challenging as the microaggression is being delivered by an attending physician. Regardless of the sexuality of the recipient of this microaggression, the statement is an affront to gay people.*
- How would you respond to this microaggression? *An attempt can be made to diffuse the situation. “Choices in sexual partners seem like a very personal and private matter.” Another option is to directly make a comment, “that can be interpreted as a homophobic statement”.*
- What strategies would you employ in your response? *When attempting to interrupt microaggressions, paying attention to tone and body language is critically important.*

**Case 3a: Supervisor to another person on your team**

We’re on a break from seeing patients and my attending starts talking about all the LGBT people in one of the classes, identifying and “outing” them without their permission. I have some queer and trans friends and know this is not only disrespectful but can be dangerous. I feel really uncomfortable that this happened.

- Why is this microaggression inappropriate? *This microaggression revolves around breaches in confidentiality and exoticizing queer and trans people. Although it may be unintentional, it is traumatizing and demeaning to its subjects.*
- How would you respond to this microaggression? *The power dynamic and hierarchy can be particularly challenging as the microaggression is being delivered by an attending physician. A direct approach may be best used here, “I’m not sure that that’s public information that you are sharing”.*
- What strategies would you employ in your response? *Empathy would be useful in this scenario. Merely bringing attention to the behavior might be helpful in the event it is unintentional.*

**Case 3b: Supervisor to another person on your team**

During my clerkship rotations, one of my team members is constantly confused with another Asian female medical student. It really upsets me when I hear attendings calling her by our other classmate’s name and asking the residents "which one is which?"

- Why is this microaggression inappropriate? *This microaggressions stems from a phenomenon called the “cross-race effect” which highlights the finding that people are better at telling apart faces of their own race than of those another race. It demeans the identity of an individual by grouping them all together by their similarities as belonging to the “Asian race.”*
- How would you respond to this microaggression? *An attempt could be made to ask a question out of curiosity such as, “Do you think all Asians look alike?” One could also reframe the statement and inquire, “How would it feel if you were always mistaken for someone else?”*
- What strategies would you employ in your response? As in all situations with a supervisor, the power dynamics are challenging in that the student may not be comfortable addressing this with their attending. Some may perceive the question, “Do you think all Asians look alike” as an opportunity to address this microaggression with humor when in actuality, it address the statement immediately.

**Case 4a: Peer to peer**

I went to an Ivy League college for undergrad, had a 4.0 GPA, and am first-author on a publication in a high-impact medical journal. Yet my fellow students seem to believe I was only accepted to this medical school because I’m black, not because of my academic achievements. This has left me feeling depressed and that my achievements aren’t valid.

- Why is this microaggression inappropriate? *This microaggressions has its roots in the notion that students underrepresented in medicine are given undeserved preferential treatment in the process of recruitment. In addition, it may have an undertone of the “zero sum game” notion that as other are accepted into medical school, it implies that “more deserving” candidates are not getting in.*
- How would you respond to this microaggression? *An attempt can be made to educate the person by noting the facts that the student is more than qualified to be in medical school based on their record of achievements in a similar way to all students. As that feeling of needing to explain oneself or justify one’s existence may not be desired, another option is to directly make a comment about how that statement could be viewed as a “racist comment” and/or acknowledging how it made that person feel.*
- What strategies would you employ in your response? *The use of “I” statements would be critical in this response as well as immediately acknowledging the inaccuracy of the statement given the facts.*

**Case 4b: Peer to peer**

I’m working on a group project and I’m the only woman in the group. Every time we divide tasks, I’m asked to do all the secretarial type work (scheduling, emailing, printing, calling) for the meetings, but the men never assign themselves these kinds of tasks. I can’t help but wonder if they think I’m less capable than they are.

- Why is this microaggression inappropriate? *This microaggressions has its roots in sexism and traditionally assigned roles of women in secretarial tasks.*
- How would you respond to this microaggression? *In this peer setting, a student could directly address the perception by noting, “I am wondering if there is a reason why the only woman in the group is being assigned to do all the secretarial tasks.” Again, as microaggressions stem from implicit bias, confronting the stereotype directly allows the peers to address the microaggression.*
- What strategies would you employ in your response? *When attempting to interrupt this microaggression, paying attention to tone and body language is critically important. It is also important to use “I” statements and to separate the comment from the behavior. For example, it would be important to not say, “it’s very sexist of you to assign these tasks to me.”*

**Case 5: Patient to you**

I’m a black woman and when I go to see a white patient during rounds, she starts screaming at me, “Why are you here?!” As I gape at her and try to come up with a response, she says, “Why would they give YOU my medical information?"

- Why is this microaggression inappropriate? *This microaggressions has its roots in racism and an assumption that the student is not being seen as a member of the healthcare team. The deliverer of the microaggression is casting judgment on the student. The power dynamic and can be difficult as the microaggression is being delivered by a patient and the patient-provider relationship is important to care.*
- How would you respond to this microaggression? *An attempt can be made to diffuse the situation by initially educating the patient the student is a part of the healthcare team and then asking the patient why they would think otherwise.”*
- What strategies would you employ in your response? *Incorporating the initial intent of PEARLS as a way to build relationships with patients may be helpful in this response as partnering with the patient to jointly solve the problem could be utilized here.*

**Case 6: Patient to someone else on your team**

I never really thought about male privilege until I was on the wards. When I walk around with my women colleagues, patients always assume they are nurses, even though they are wearing white coats. That never happens to me.

- Why is this microaggression inappropriate? *This is an awareness of a microaggression that commonly happens to medical students (residents and faculty) that identify as female.*
- How would you respond to this microaggression? *This student should be empowered to not only be aware of this microaggression but to be an ally to his female colleagues on the wards. When these situations occur, male students can respond by highlighting their awareness and when the situation presents itself, to educate others.*
- What strategies would you employ in your response? *N/A (as this is not a microaggression)*

**Case 7: Faculty to faculty**

I am a part of an advisory board where I am the only person of color, everybody else is white. We have been tasked with advising the hospital on all things related to the patient experience. This board will likely shape some policy and even the aesthetics of the hospital. I mentioned that the board does not represent the diverse population we serve and that we should invite more diverse folks to our future meetings. It’s been 6 months and nothing has changed.

- Why is this microaggression inappropriate? *This microaggressions has its roots in the minority tax that comes from physicians underrepresented in medicine being viewed as the representative spokesperson for all areas related to diversity, equity and inclusion.*
- How would you respond to this microaggression? *An attempt can be made to remind the organizers that although you will share your input, that you are not able to share the perspective of all the various constituents that comprise the patient population and that if the goals of the board are to be inclusive, then additional representation must be sought.*
- What strategies would you employ in your response? *Strategies include an understanding of the organizational culture inherent to the institution. It would be important to highlight the mission and core values and your efforts to align with them in the response.*

# Pairs Activity : Statements

Imagine you overhear the following statements on the wards. Work in pairs to discuss the following:

Why is this a microaggression?

How would you respond in the moment?

1. A patient says, “Oh good, transport is here.” The patient is referring to a doctor that identifies as black.
2. A student says, “Ugh, they treat us like slaves.”
3. A lecturer says, “I don’t see race, I treat all of my patients the same.”
4. A resident asks your patient, who’s a woman, “Do you have a husband?”
5. A provider says, “What do I do with it?” when referring to a transgender patient.
6. A patient, referring to another patient, says, “Ugh, I wish these people would really learn how to speak English. We’re in America.”

# Pairs Activity : Microaggressive Statements

Imagine you overhear/witness the following statements on the wards. Work in pairs to discuss the following:

Why is this a microaggression?

How would you respond in the moment?

1. ***A patient says, “Oh good, transport is here.” The patient is referring to a doctor that identifies as black.***
   - Why is this a microaggression? This stems from a racist assumption that a black person is more likely to be viewed as a part of the ancillary staff as opposed to a physician.
   - How would you respond in the moment? One could respond by saying, “Actually, he is not the transporter. He is Dr. X and he is the senior resident. I am wondering what made you assume that he was the transporter.”
2. ***A student says, “Ugh, they treat us like slaves.”***
   - Why is this a microaggression? This stems from a racist assumption that that it is meaningless to use the term “slave,” without noting how offensive the comment is given the history of slavery.
   - How would you respond in the moment? One could respond by saying, “Considering the history of slavery in this country, it makes me uncomfortable to hear you say that.”
3. ***A lecturer says, “I don’t see race, I treat all of my patients the same.”***
   - Why is this a microaggression? This is a microinvalidation which nullifies the experiences of a person of color such that their experiences are invisible without taking into account the factual existence of health disparties and inequities that abound.
   - How would you respond in the moment? One could respond by aksing with genuine curiosity about how to interpret the impact of implicit bias on clinical decision making if one does not see the race of the patients they are treating.
4. ***A resident asks your patient, who’s a woman, “Do you have a husband?”***
   - Why is this a microaggression? This stems from a heterosexist assumption that because she is a woman, that her partner must be a man.
   - How would you respond in the moment? One could respond by saying, “If the question is relevant, it is more inclusive to ask about someone’s partner as opposed to assuming that we know to whom someone is physically attracted.”
5. ***A provider says, “What do I do with it?” when referring to a transgender patient.***

- Why is this a microaggression? This is an assumption that a transgender patient doesn’t merit the same respectful pronouns as a cisgender person. This is transphobic.
  - How would you respond in the moment? One could respond by saying, “Asking all patients for their preferred gender pronouns will help in our attempt to be patient-centered and provide equitable care for all.”

1. ***A patient, referring to another patient, says, “Ugh, I wish these people would really learn how to speak English. We’re in America.”***
   - Why is this a microaggression? This stems from xenophobia and highlights the anyone who speaks another language is not American.
   - How would you respond in the moment? One could respond by asking “Many languages are spoken in America. I’m wondering why someone who doesn’t speak English makes them less American.”

Infographic: Intent & Impact

IMPACT

behavior

intention

intended consequences

unintended consequences

Author created.

The Regents of the University of California. Intent and Impact: A Tool for Recognizing Impact. University of California San Francisco Diversity Tools for Department Chairs and Deans website. <https://diversity.ucsf.edu/sites/diversity.ucsf.edu/files/Tools%20for%20Department%20Chairs%20and%20Deans.pdf>. Accessed September 7, 2016.

Infographic: Interrupting Microaggressions

Use "I" statements describing how the action affected you or refer to the action indirectly. For example, "When ____ happened, I felt ____.”

Ask questions about the behavior using "How..." or "What made you..." rather than "Why..." as this can make folks defensive.

Separate the person from the behavior. For example, "that could be perceived as a racist remark," rather than "You're racist."

Note your tone of voice, body language, etc. when responding. What is your intention and goal for your outcome? How can your tone and body language help you acheive this?

Ack!

You just witnessed a microaggression.

Now what?

Author created.

The Regents of the University of California. Tool: Interrupting Microaggressions. University of California San Francisco Diversity Tools for Department Chairs and Deans website. <https://diversity.ucsf.edu/sites/diversity.ucsf.edu/files/Tools%20for%20Department%20Chairs%20and%20Deans.pdf>. Accessed September 7, 2016.

| **MICROAGGRESSION**  **EXAMPLE AND THEME** | **THIRD PARTY**  **INTERVENTION EXAMPLE** | **COMMUNICATION APPROACH** |
| --- | --- | --- |
| **Alien in One’s Own Land**  To a Latino American: “Where are you from?”  **Ascription of Intelligence**  To an Asian person, “You’re all good in math, can you help me with this problem?”  **Color Blindness**  “I don’t believe in race.” | “I’m just curious. What makes you ask that?”  “I heard you say that all Asians are good in math. What makes you believe that?”  “So, what do you believe in? Can you elaborate?” | **INQUIRE**  Ask the speaker to elaborate. This will give you more information about where s/he is coming from, and may also help the speaker to become aware of what s/he is saying.  **KEY PHRASES:**  “Say more about that.”  “Can you elaborate on your point?”  “It sounds like you have a strong opinion about this. Tell me why.”  “What is it about this that concerns you the most?” |
| **Myth of Meritocracy** “Everyone can succeed in this society, if they work hard enough.”  **Pathologizing Cultural Values/Communication Styles**  Asking a Black person: “Why do you have to be so loud/animated? Just calm  down.” | “So you feel that everyone can succeed in this society if they work hard enough. Can you give me some examples?”  “It appears you were uncomfortable when said that. I’m thinking that there are many styles to express ourselves. How we can honor all styles of expression—can we talk about  that?” | **PARAPHRASE/REFLECT**  Reflecting in one’s own words the essence of what the speaker has said. Paraphrasing demonstrates understanding and reduces defensiveness of both you and the speaker. Restate briefly in your own words, rather than simply parroting the speaker. Reflect both content and feeling whenever possible.  **KEY PHRASES:**  “So, it sounds like you think…” “You’re saying…You believe…” |
| **Second-Class Citizen**  You notice that your female colleague is being frequently interrupted during a committee meeting.  **Pathologizing Cultural Values/Communication Styles**  To a woman of color: “I would have never guessed that you  were a scientist.” | Responder addressing the group: “ brings up a good point. I didn’t get a chance to hear all of it. Can repeat it?”  “I’m wondering what message this is sending her. Do you think you would have said this to a white male?” | **REFRAME**  Create a different way to look at a situation.  **KEY PHRASES:**  **“**What would happen if….”  “Could there be another way to look at this…” “Let’s reframe this…”  “How would you feel if this happened to your_ ” |
| **Second-Class Citizen**  Saying “You people….”  **Use of Heterosexist Language**  Saying “That’s so gay.” | “I was so upset by that remark that I shut down and couldn’t hear anything else.”  “When I hear that remark, I’m offended too, because I feel that it marginalizes an entire group of people that I work with.” | **USE IMPACT AND “I” STATEMENTS**  A clear, nonthreatening way to directly address these issues is to focus on oneself rather than on the person. It communicates the impact of a situation while avoiding blaming or accusing the other and reduces defensiveness.  **KEY PHRASES:**  “I felt (*feelings*) when you said or did  (*comment or behavior*), and it  (*describe the impact on you*).” |
| **Second-Class Citizen**  A woman who is talked over.  Making a racist, sexist or homophobic joke. | She responds: “I would like to participate, but I need you to let me finish my thought.”  “I didn’t think this was funny. I would like you to stop.” | **USE PREFERENCE STATEMENTS**  Clearly communicating one’s preferences rather than stating them as demands or having others guess what is needed.  **KEY PHRASES:**  “What I’d like is…”  “It would be helpful to me if….” |

**Tool: Interrupting Microaggressions**

## Tool: Interrupting Microaggressions

| **MICROAGGRESSION**  **EXAMPLE AND THEME** | **THIRD PARTY**  **INTERVENTION EXAMPLE** | **COMMUNICATION APPROACH** |
| --- | --- | --- |
| **Color Blindness**  “When I look at you, I don’t see color.”  **Myth of Meritocracy**  “Of course he’ll get tenure, even though he hasn’t published much—he’s Black!” | “So you don’t see color. Tell me more about your perspective. I’d also like to invite others to weigh in.”  “So you believe that will get tenure just because of his race.  Let’s open this up to see what others think.” | **RE-DIRECT**  Shift the focus to a different person or topic. (Particularly helpful when someone is asked to speak for his/her entire race, cultural group, etc.) **KEY PHRASES:**  “Let’s shift the conversation…”  “Let’s open up this question to others….” |
| **Myth of Meritocracy**  In a committee meeting: “Gender plays no part in who we hire.”  “Of course she’ll get tenure, even though she hasn’t published much—she’s Native American!”  **Second-Class Citizen**  In class, an instructor tends to call on male students more frequently than female ones. | “How might we examine our implicit bias to ensure that gender plays no part in this and we have a fair process? What do we need to be aware of?”  “How does what you just said honor our colleague?”  “What impact do you think this has on the class dynamics? What would you need to approach this situation differently next time?” | **USE STRATEGIC QUESTIONS**  It is the skill of asking questions that will make a difference. A strategic question creates motion and options, avoids “why” and “yes or no” answers, is empowering to the receiver, and allows for difficult questions to be considered.  Because of these qualities, a strategic question can lead to transformation. Useful in problem- solving, difficult situations, and change efforts. **KEY PHRASES:**  “What would allow you…”  “What could you do differently….”  “What would happen if you considered the impact on…” |
| **Traditional Gender Role Prejudicing and Stereotyping** In the lab, an adviser asks a female student if she is planning to have children while in postdoctoral training. | To the adviser: “I wanted to go back to a question you asked    yesterday about her plans for a family. I‘m wondering what made you ask that question and what message it might have sent to her.”  To the student: “I heard what your advisor said to you yesterday. I thought it was inappropriate and I just wanted to check in with you.” | **REVISIT**  Even if the moment of a microaggression has passed, go back and address it. Research indicates that an unaddressed microaggression can leave just as much of a negative impact as the microaggression itself.  **KEY PHRASES:**  “I want to go back to something that was brought up in our conversation/meeting/class ….”  “Let’s rewind minutes…” |
| **CONSIDERATIONS:**   - The communication approaches are most effective when used in combination with one another, e.g., using impact and preference statements, using inquiry and paraphrasing together, etc - Separate the person from the action or behavior. Instead of saying “you’re racist”, try saying “that could be perceived as a racist remark.” Being called a racist puts someone on the defensive and can be considered “fighting words.” - Avoid starting questions with “Why”—it puts people on the defensive. Instead try “how” “what made you …..” - When addressing a microaggression, try to avoid using the pronoun “you” too often—it can leave people feeling defensive and blamed. Use “I” statements describing the impact on you instead or refer to the action indirectly, e.g., “when was said…” or “when happened…”   How you say it is as critical as what you say, e.g., tone of voice, body language, etc. The message has to be conveyed with respect for the other person, even if one is havina strong negative reaction to what’s been said. So it is helpful to think about your intention when interrupting a microaggression—e.g., do you want that person to understand the impact of his/her action, or stop his/her behavior, or make the person feel guilty, etc. Your intention and the manner in which you execute your intention make a difference.   - Sometimes humor can defuse a tense situation. | | |

Adapted from Kenney, G. (2014). *Interrupting Microaggressions*, College of the Holy Cross, Diversity Leadership & Education. Accessed on-line, October 2014. Kraybill, R. (2008). “*Cooperation Skills,”* in Armster, M. and Amstutz, L., (Eds.), *Conflict Transformation and Restorative Justice Manual,* 5^th^ Edition, pp. 116-117. LeBaron, M. (2008). “*The Open Question,”* in Armster, M. and Amstutz, L., (Eds.), *Conflict Transformation and Restorative Justice Manual,* 5^th^ Edition, pp. 123-124. Peavey, F. (2003). “*Strategic Questions as a Tool for Rebellion,*” in Brady, M., (Ed.), *The Wisdom Listening, Boston: Wisdom Publ., pp. 168-189.*

Author Created

The Regents of the University of California. Tool: Interrupting Microaggressions. University of California San Francisco Diversity Tools for Department Chairs and Deans website. <https://diversity.ucsf.edu/sites/diversity.ucsf.edu/files/Tools%20for%20Department%20Chairs%20and%20Deans.pdf>. Accessed September 7, 2016.
