# Supplementary material for: Interrupting Microaggressions in Health Care Settings: A Guide for Teaching Medical Students
Source: MedEdPORTAL. 2020 Jul 31;16:10969. doi: 10.15766/mep_2374-8265.10969 (PMC7394346; doi:10.15766/mep_2374-8265.10969)
Supplement: Supplementary file 1 — Preworkshop Survey.docxFacilitator Guide.docxWorkshop Presentation.pptxFaculty Development Agenda.docxPostworkshop Evaluation Form - Students.docxPostworkshop Debriefing Questions - Faculty.docx [file mep_2374-8265.10969-s001.zip › C. Workshop Presentation.pptx]

## Slide 1
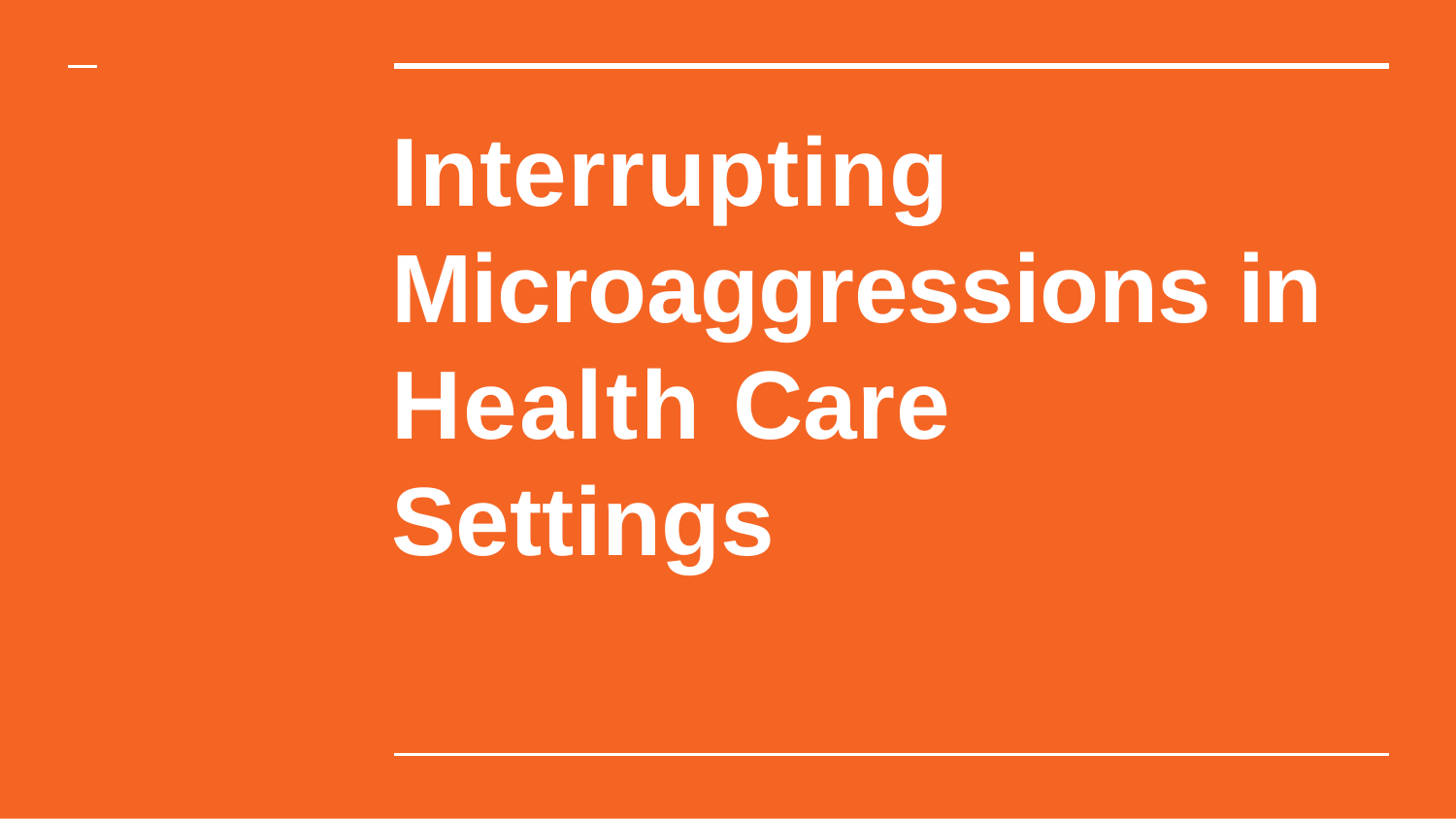

# Interrupting Microaggressions in Health Care Settings

## Slide 2
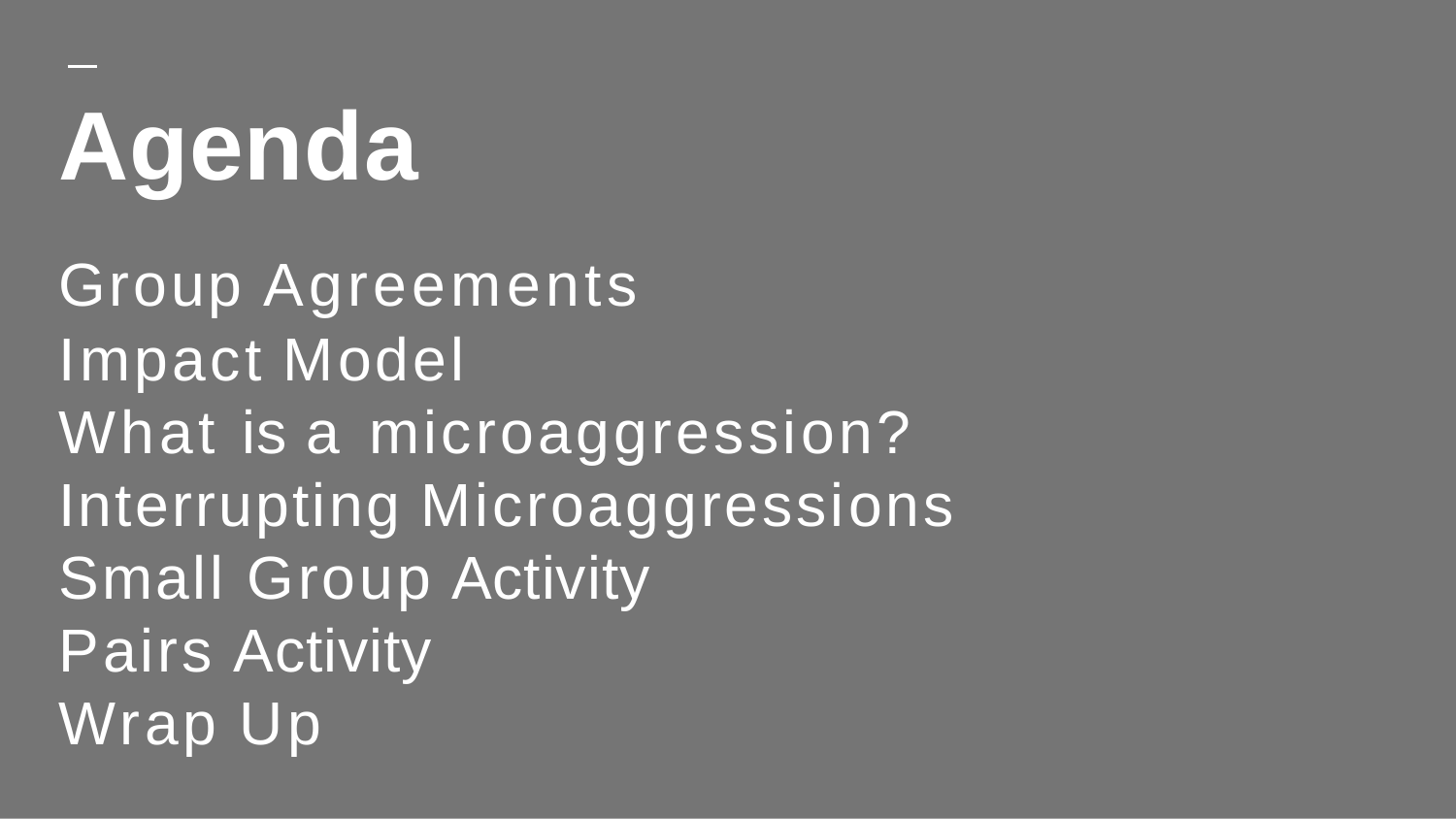

# Agenda
Group Agreements
Impact Model
What is a microaggression? Interrupting Microaggressions
Small Group Activity
Pairs Activity
Wrap Up

## Slide 3
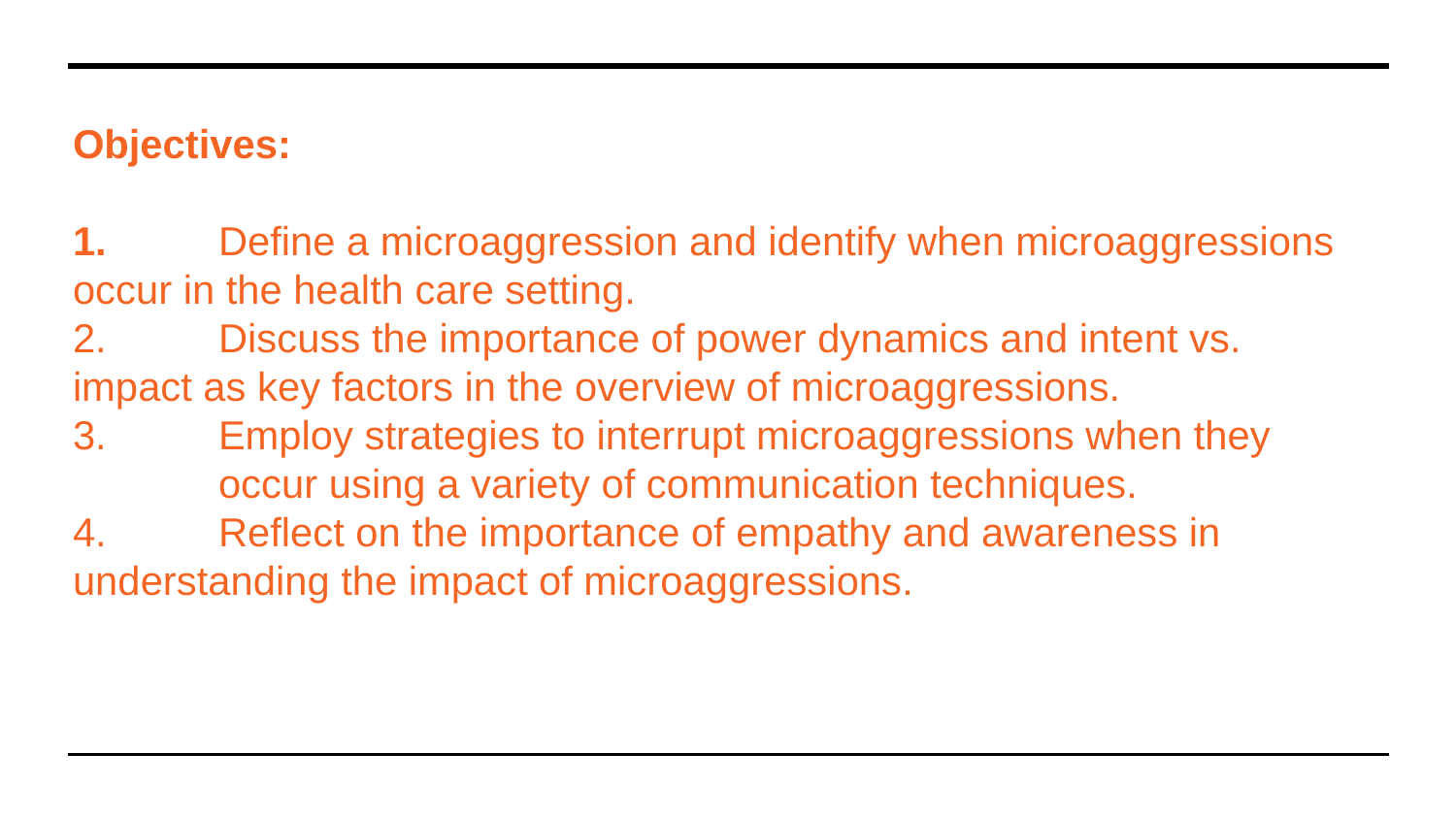

# Objectives: 1. 	Define a microaggression and identify when microaggressions 	occur in the health care setting. 2. 	Discuss the importance of power dynamics and intent vs. 	impact as key factors in the overview of microaggressions. 3. 	Employ strategies to interrupt microaggressions when they 		occur using a variety of communication techniques. 4. 	Reflect on the importance of empathy and awareness in 	understanding the impact of microaggressions.

## Slide 4
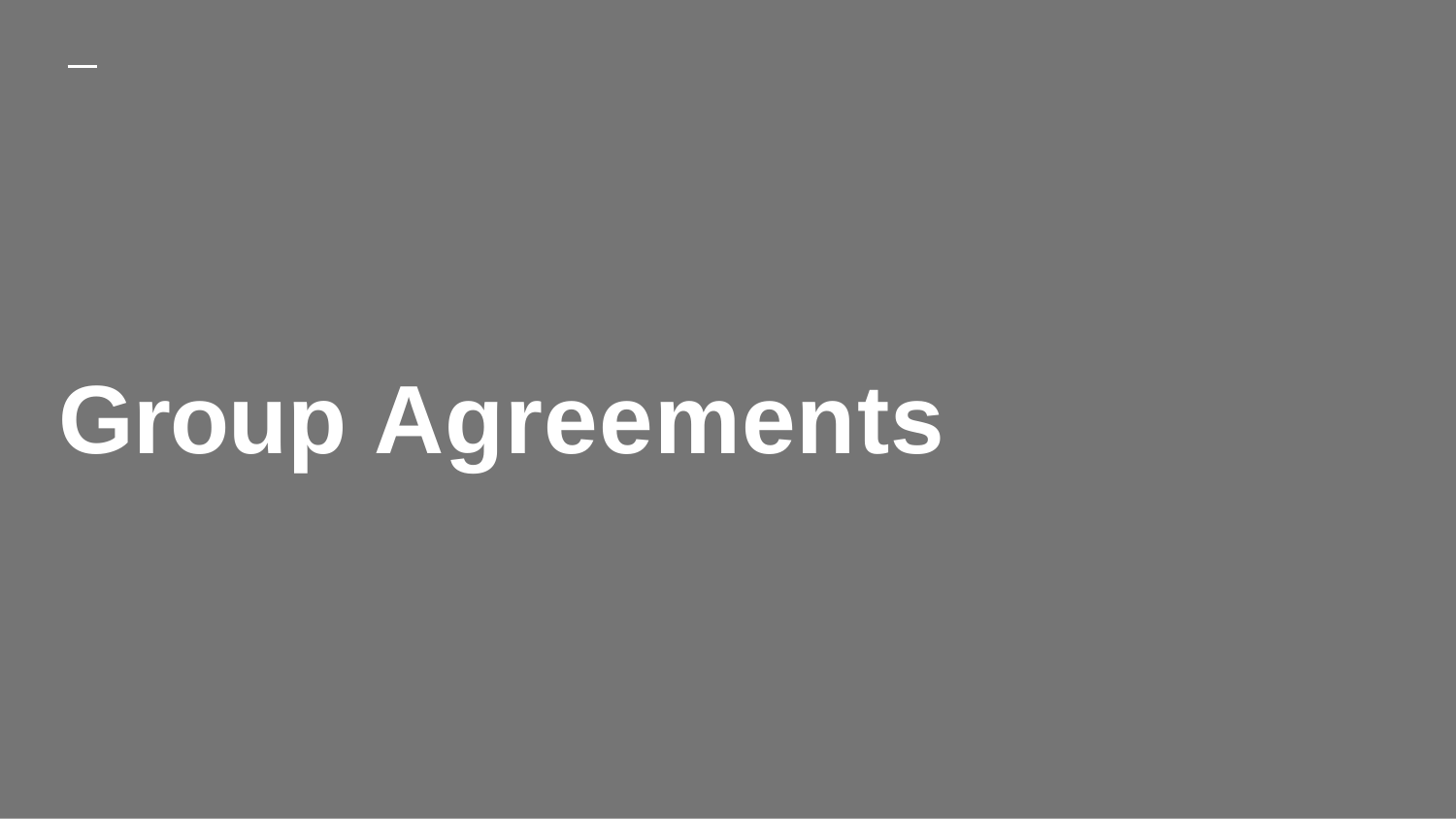

# Group Agreements

## Slide 5
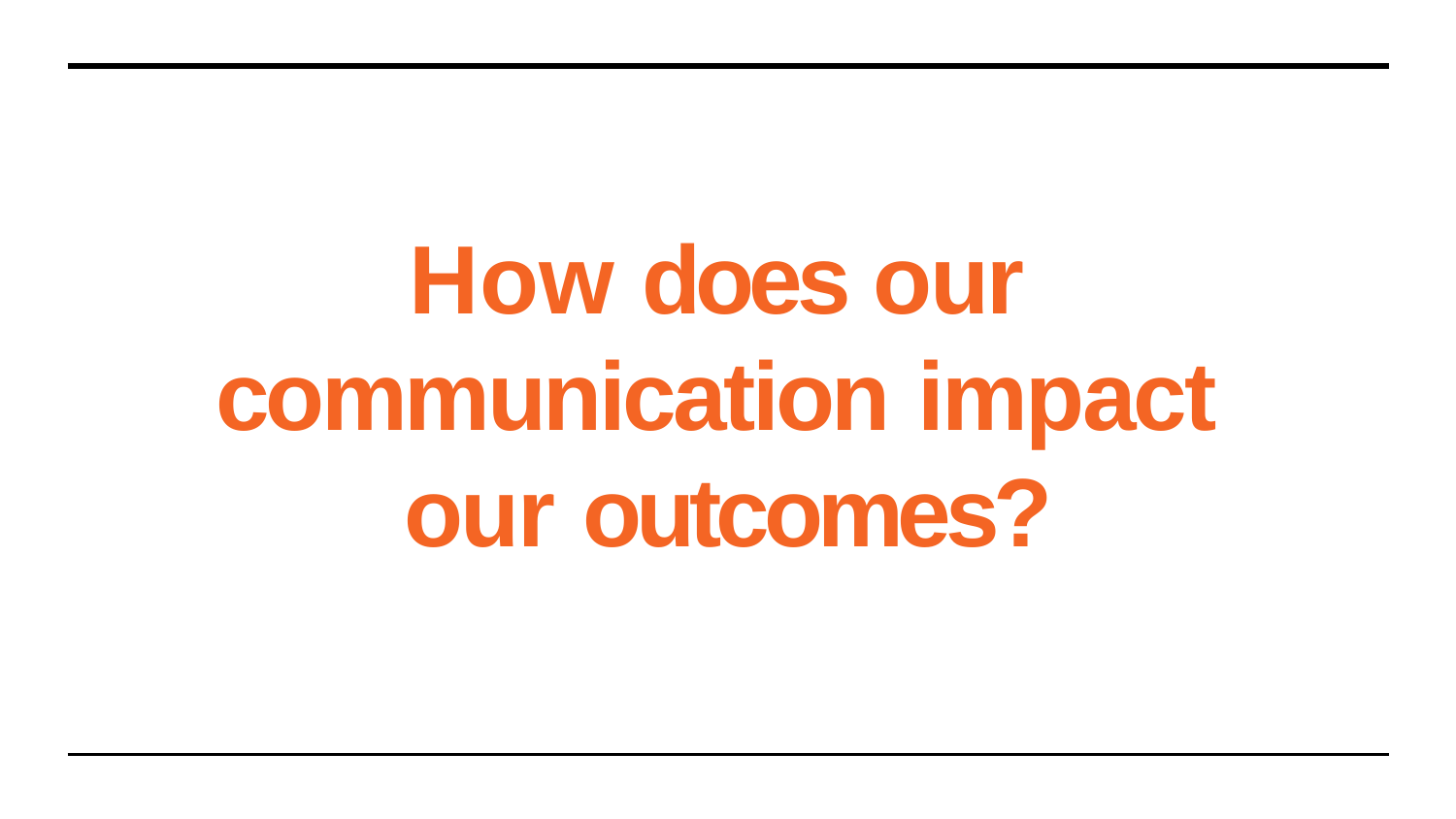

# How does our communication impact our outcomes?

## Slide 6
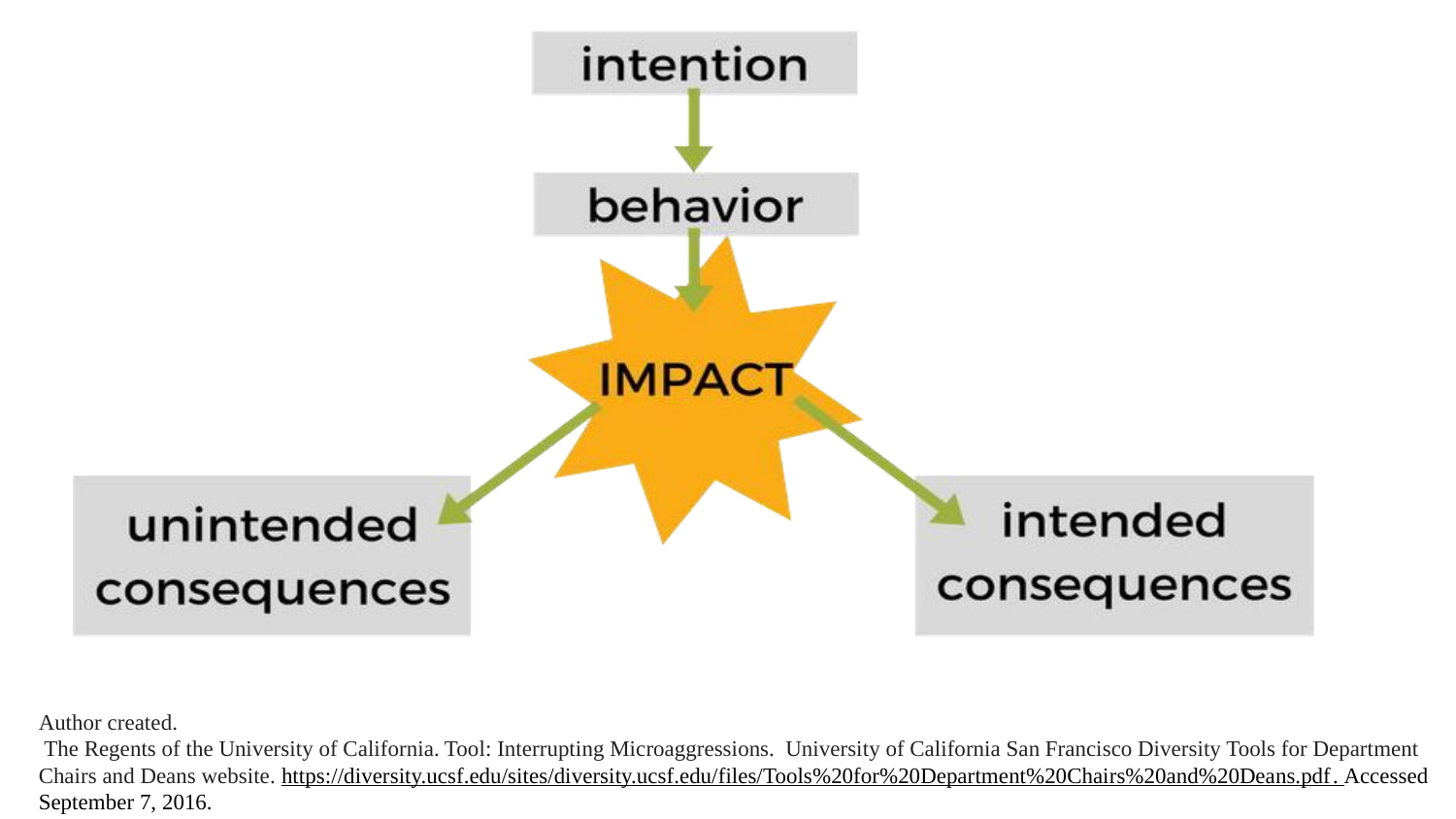

Author created.
 The Regents of the University of California. Tool: Interrupting Microaggressions. University of California San Francisco Diversity Tools for Department Chairs and Deans website. https://diversity.ucsf.edu/sites/diversity.ucsf.edu/files/Tools%20for%20Department%20Chairs%20and%20Deans.pdf. Accessed September 7, 2016.

## Slide 7
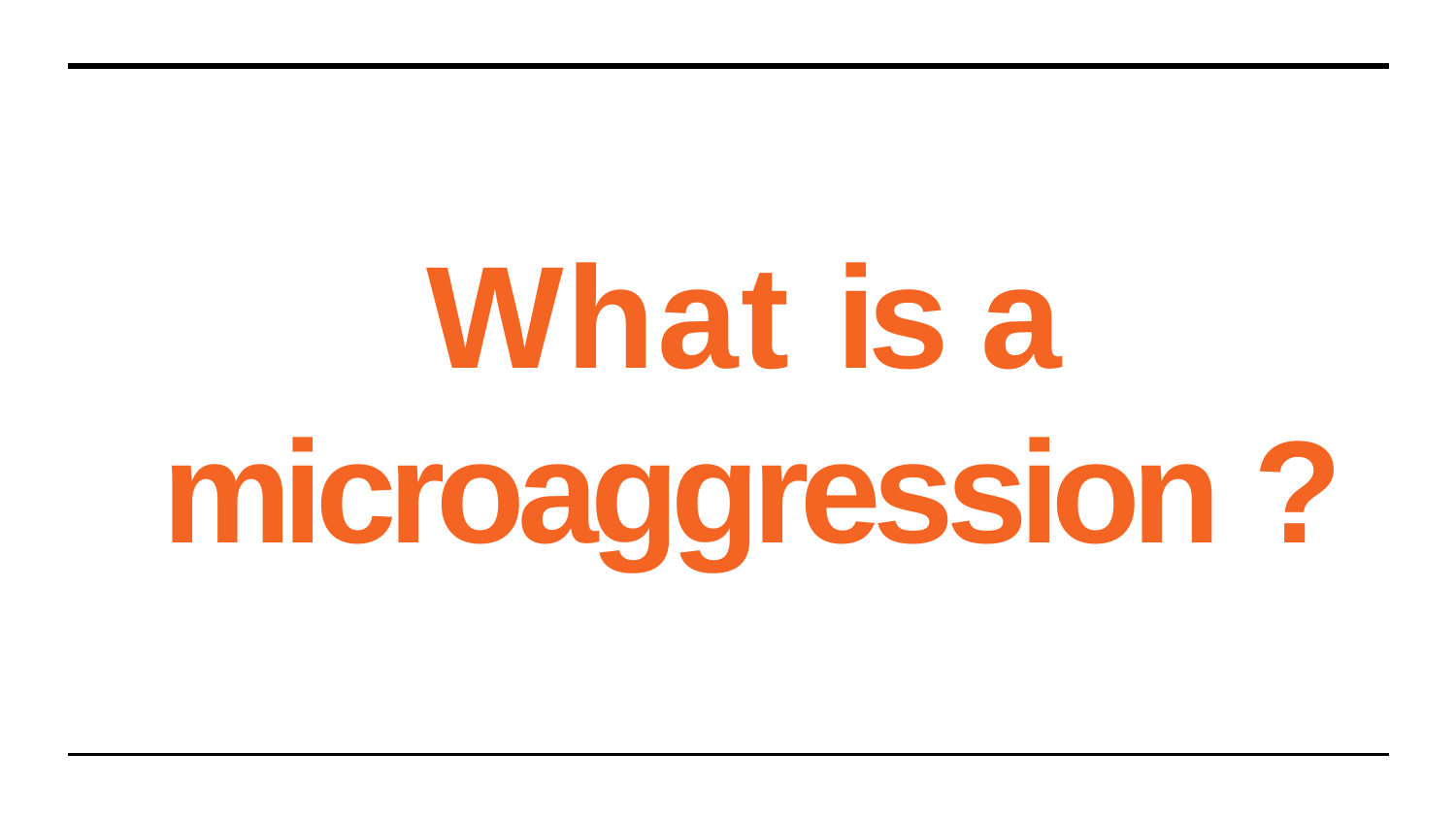

# What is a microaggression ?

## Slide 8
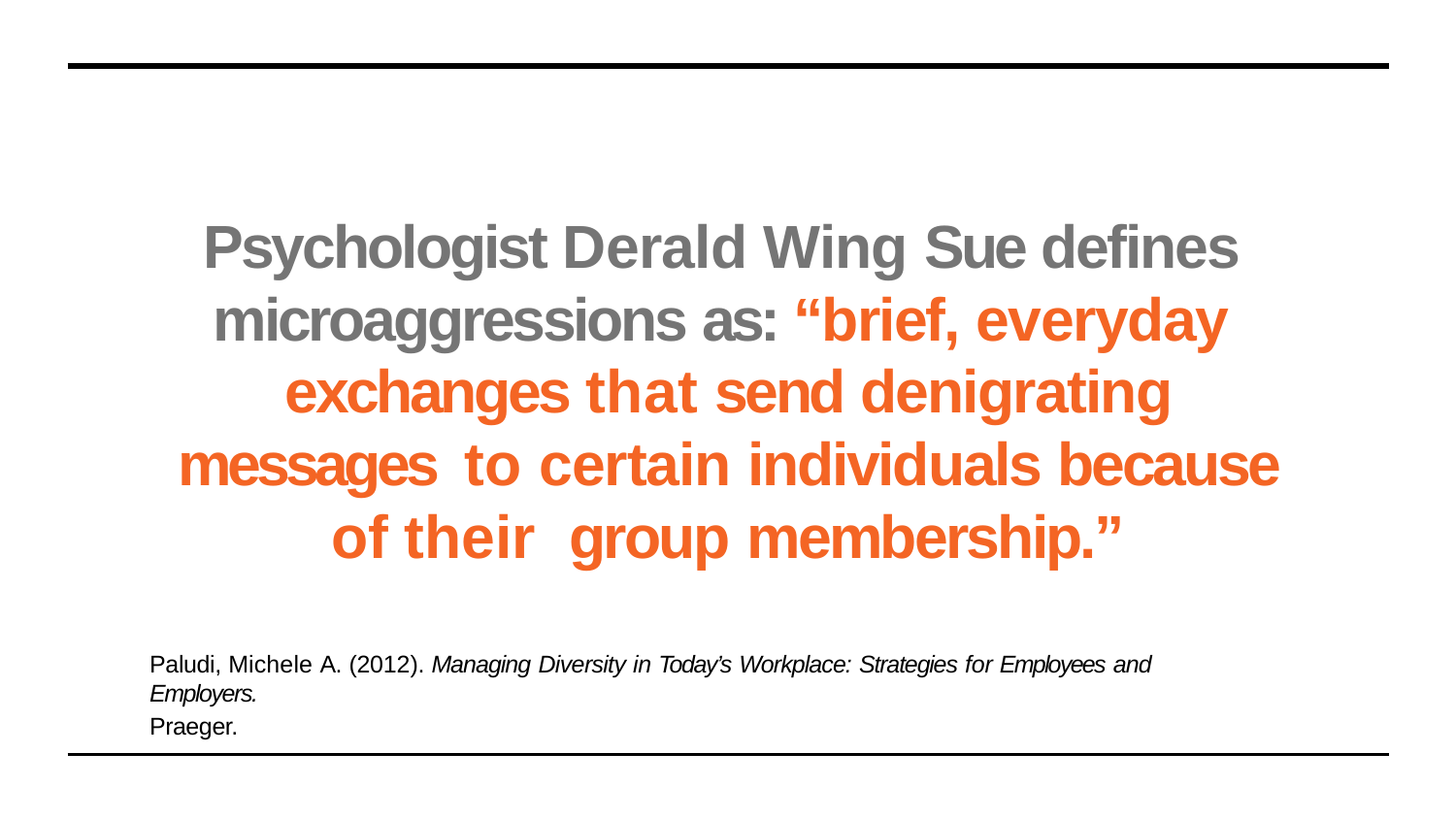

# Psychologist Derald Wing Sue defines microaggressions as: “brief, everyday exchanges that send denigrating messages to certain individuals because of their group membership.”
Paludi, Michele A. (2012). Managing Diversity in Today’s Workplace: Strategies for Employees and Employers.
Praeger.

## Slide 9
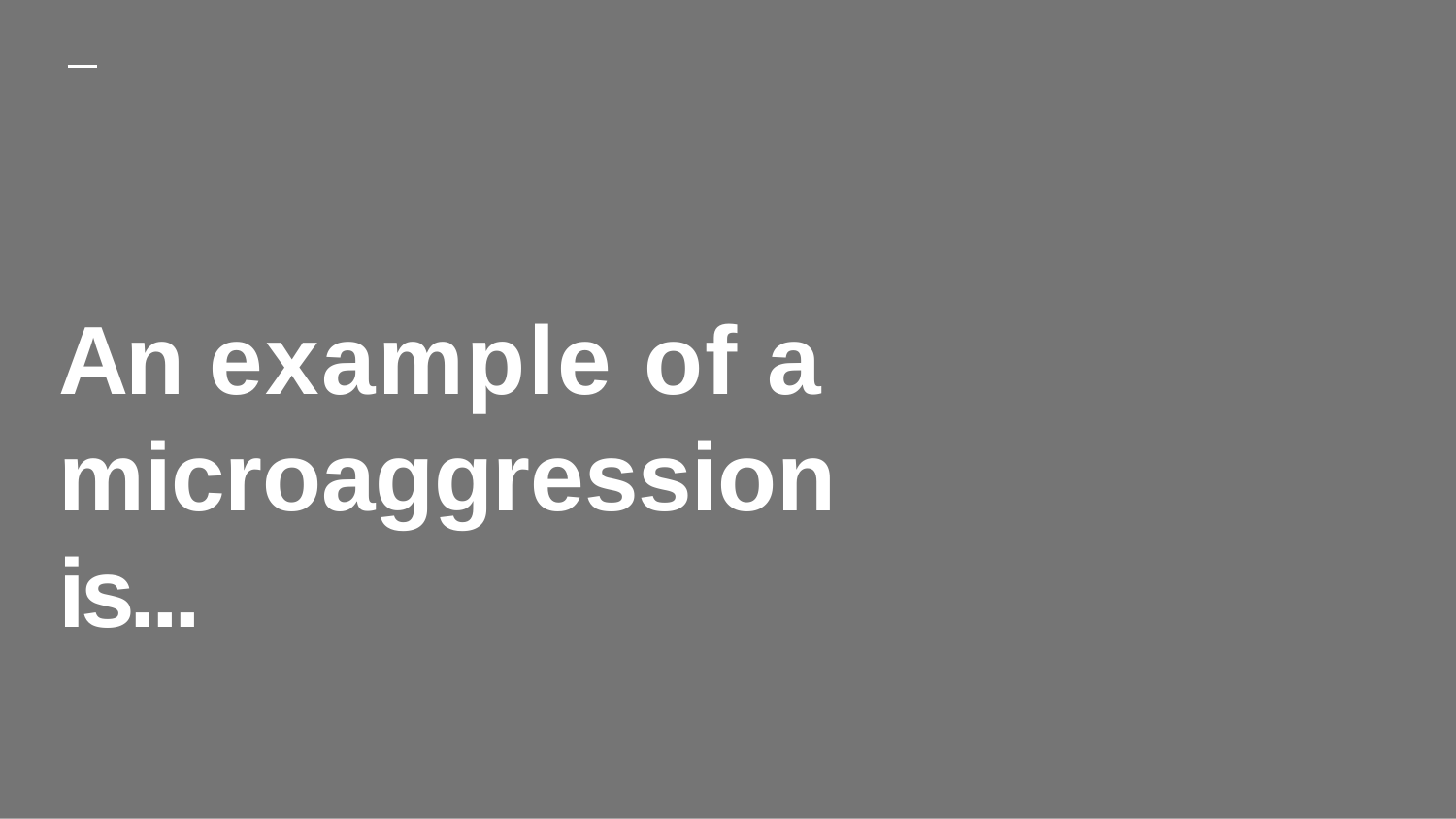

# An example of a microaggression is...

## Slide 10
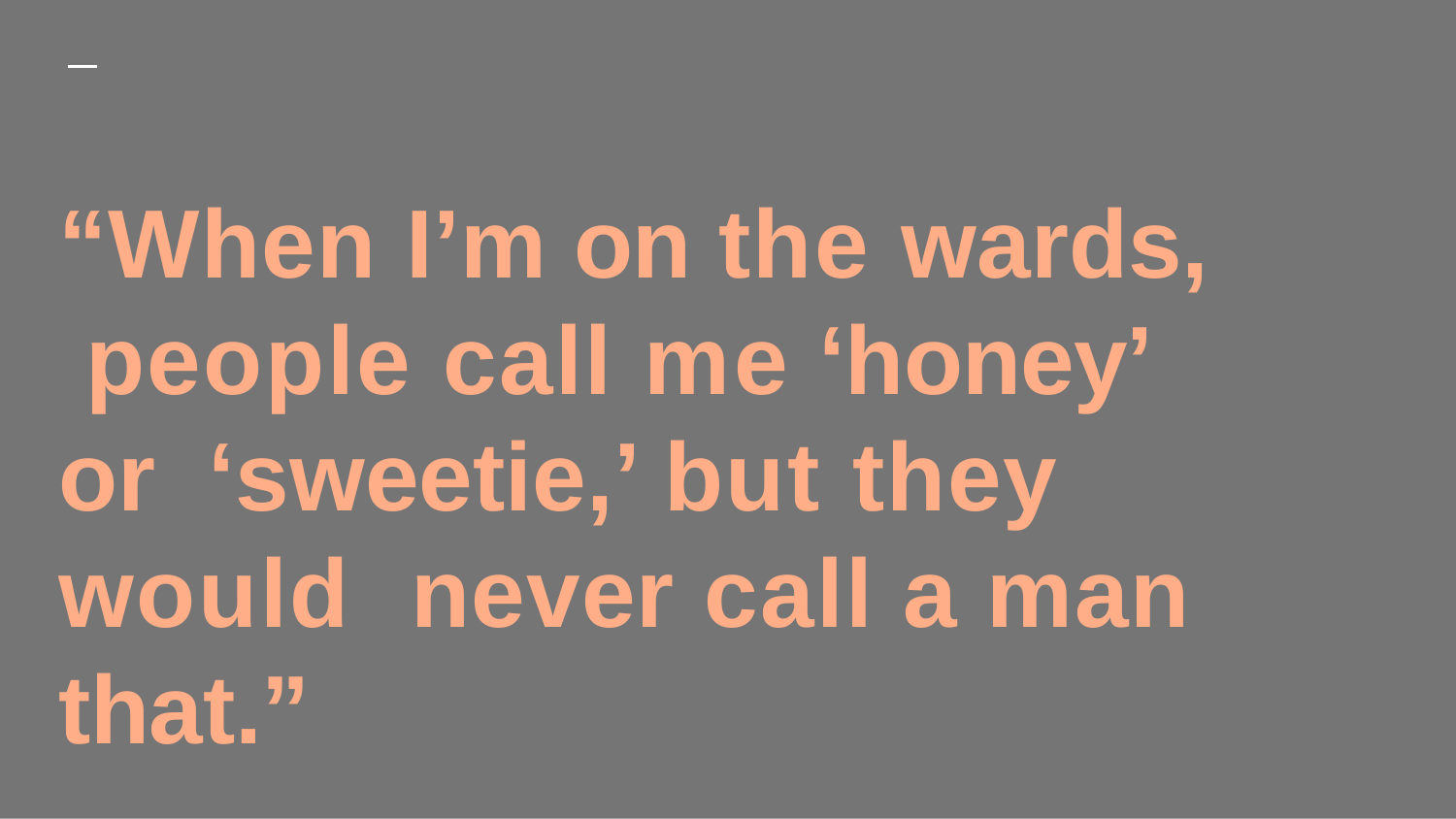

“When I’m on the wards, people call me ‘honey’ or ‘sweetie,’ but they would never call a man that.”

## Slide 11
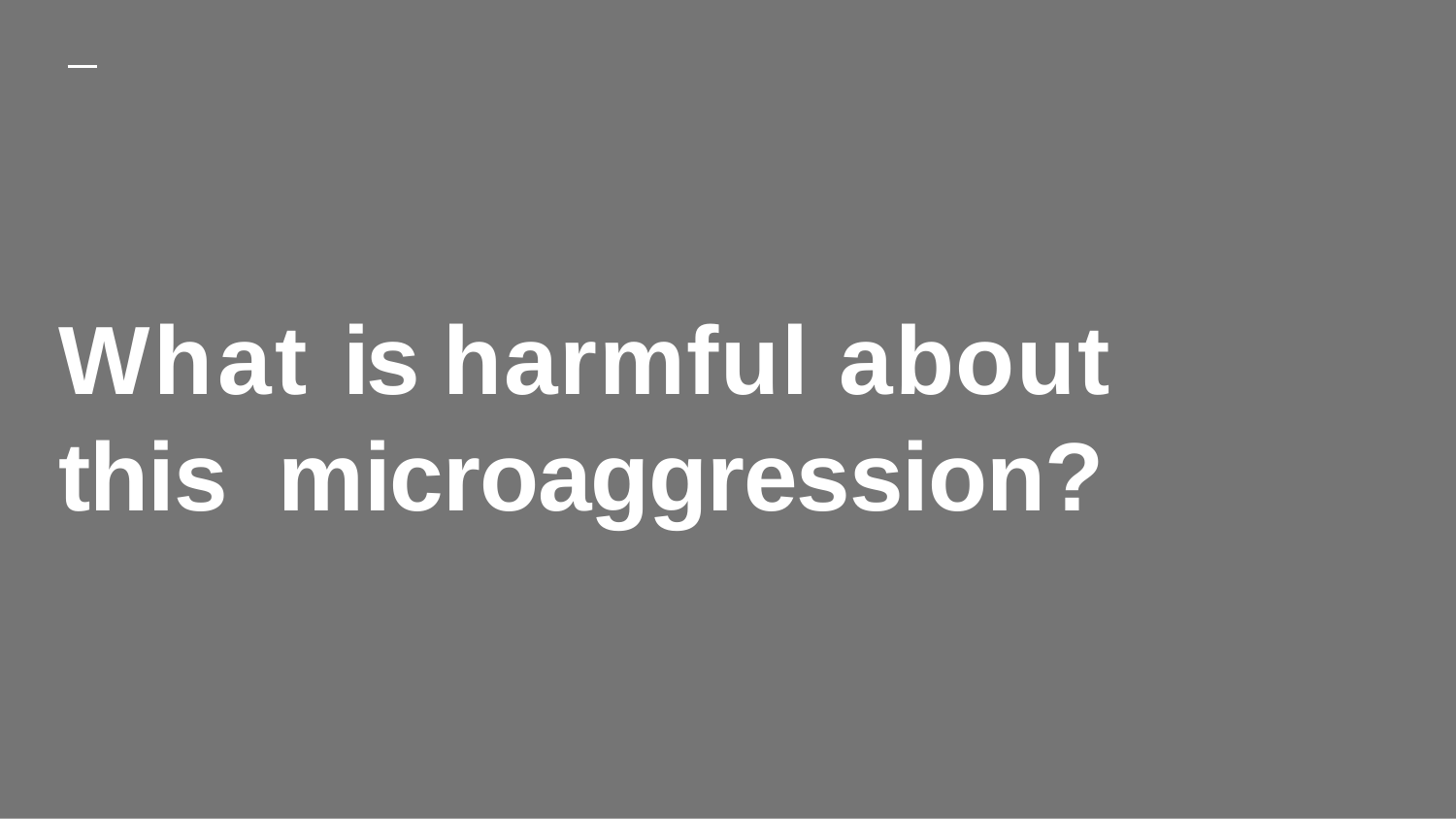

# What is harmful about this microaggression?

## Slide 12
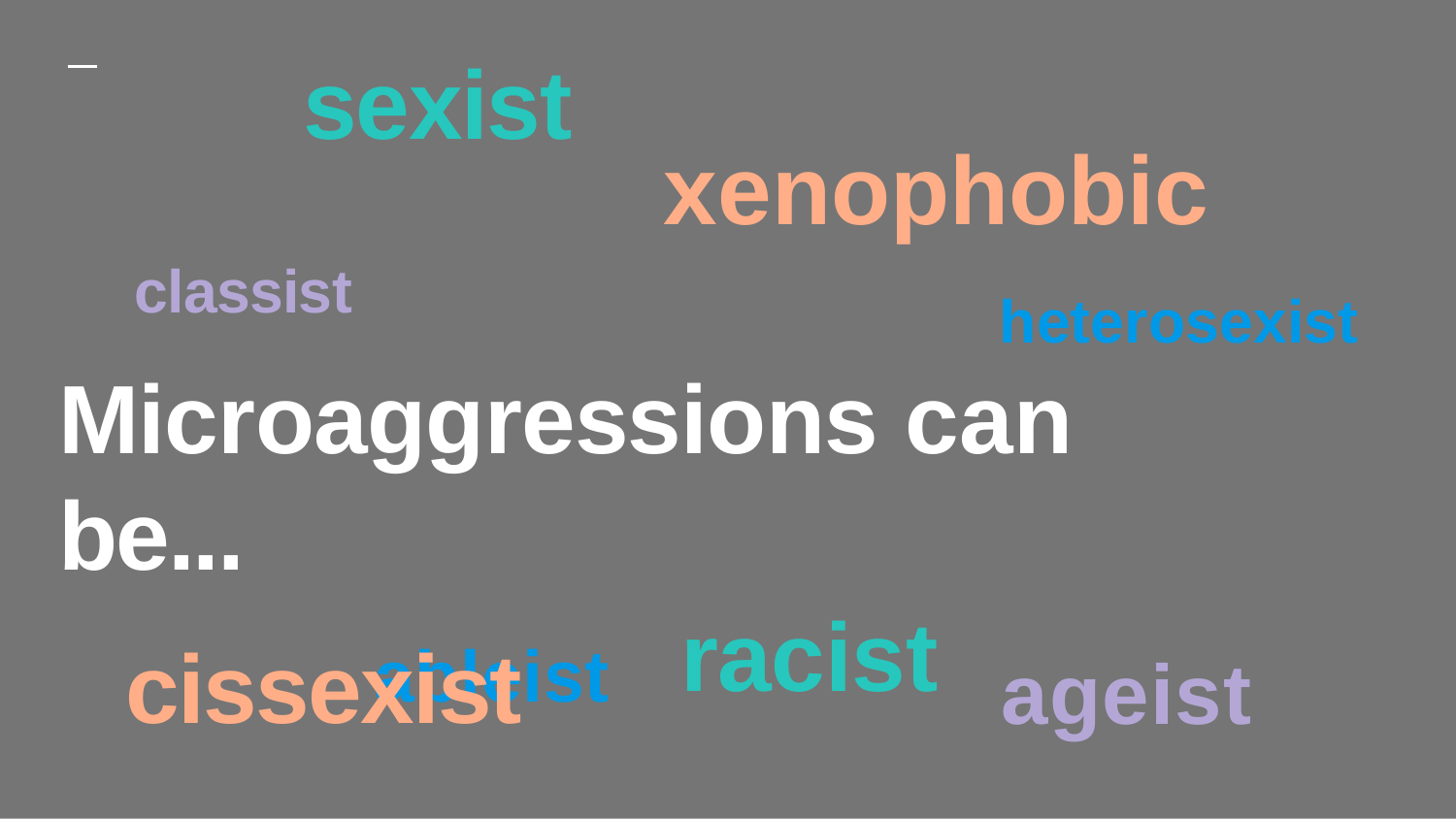

# sexist
xenophobic
heterosexist
classist
Microaggressions can be...
ableist
racist	ageist
cissexist

## Slide 13
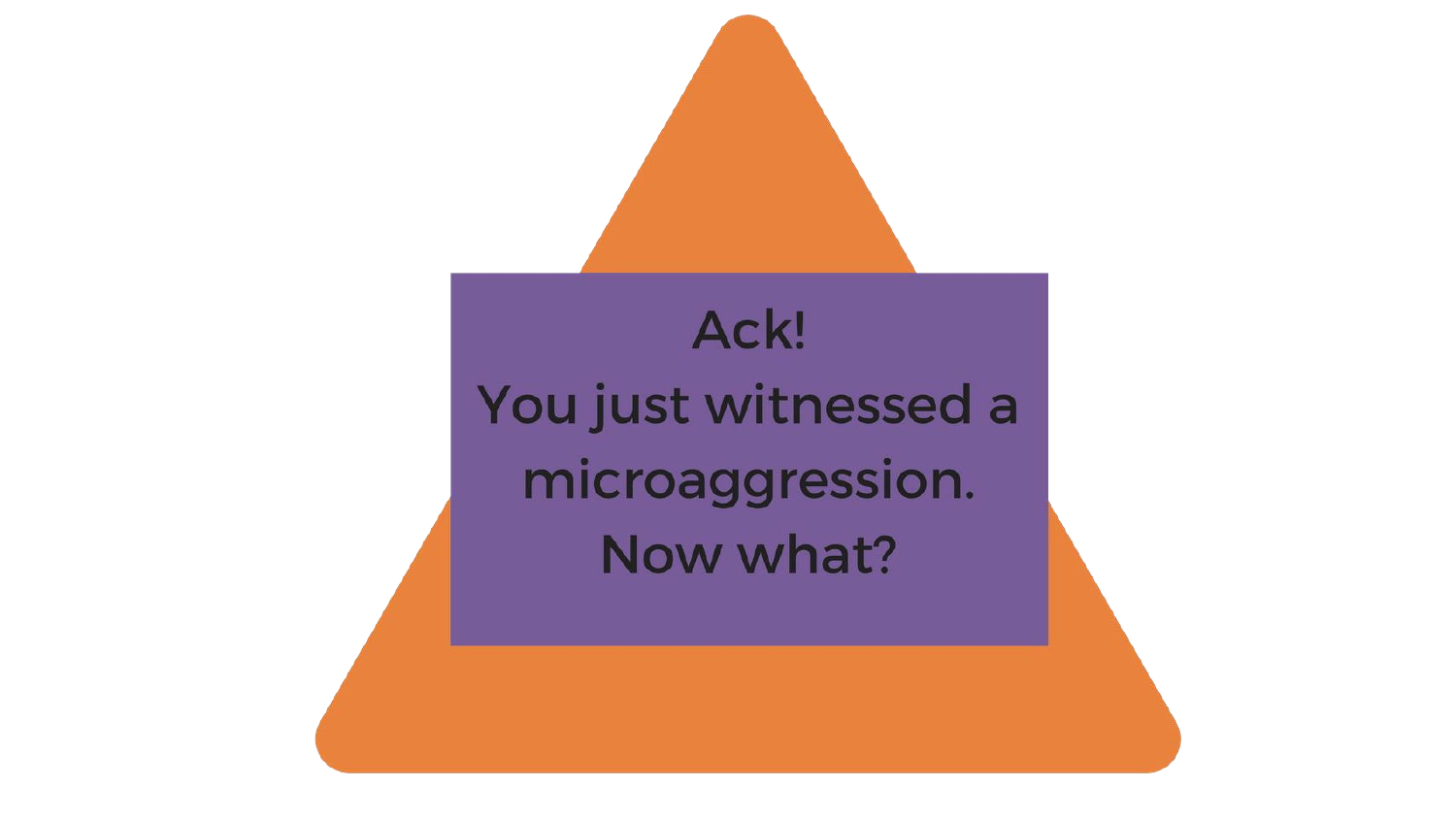

## Slide 14
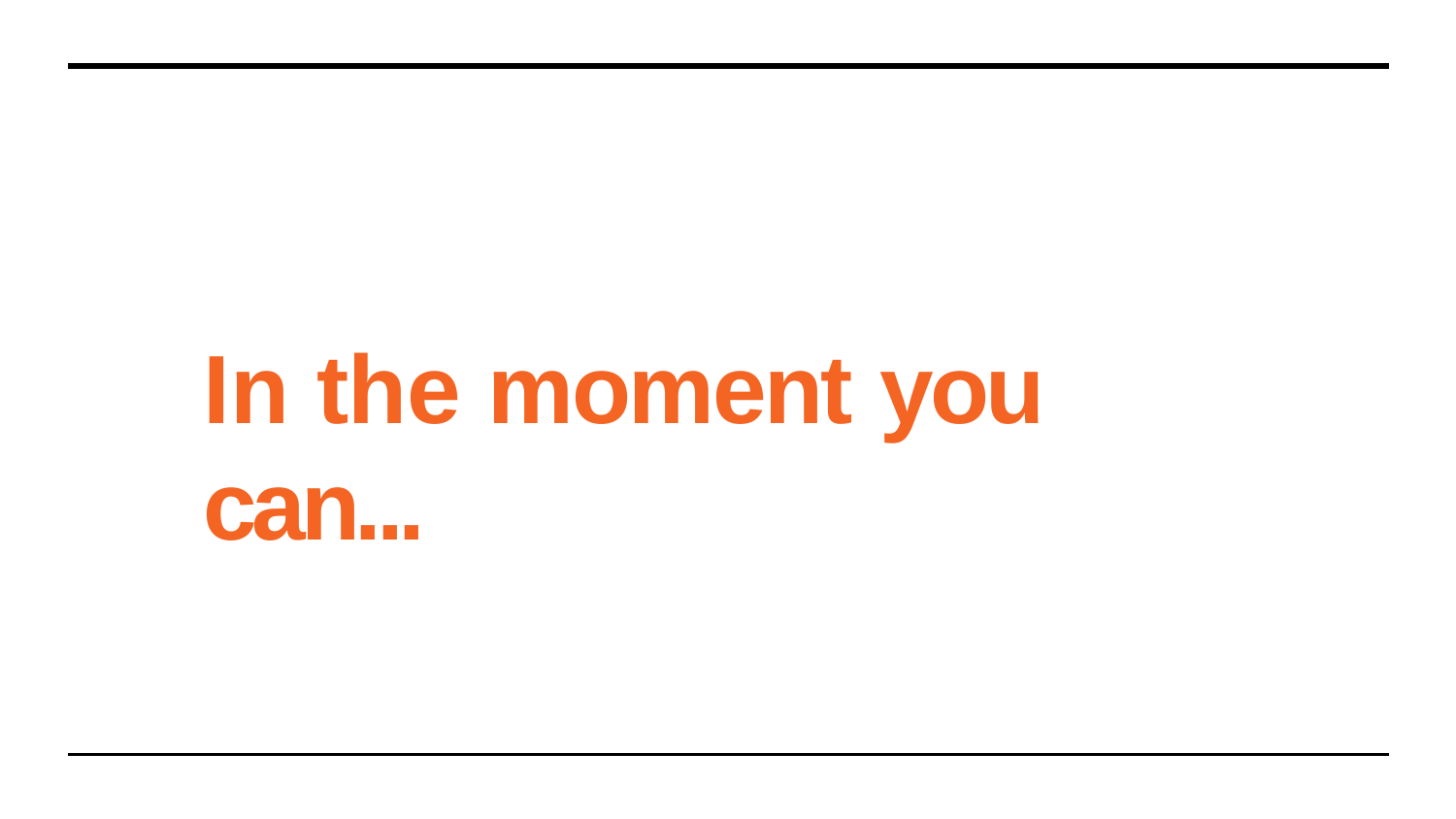

# In the moment you can...

## Slide 15
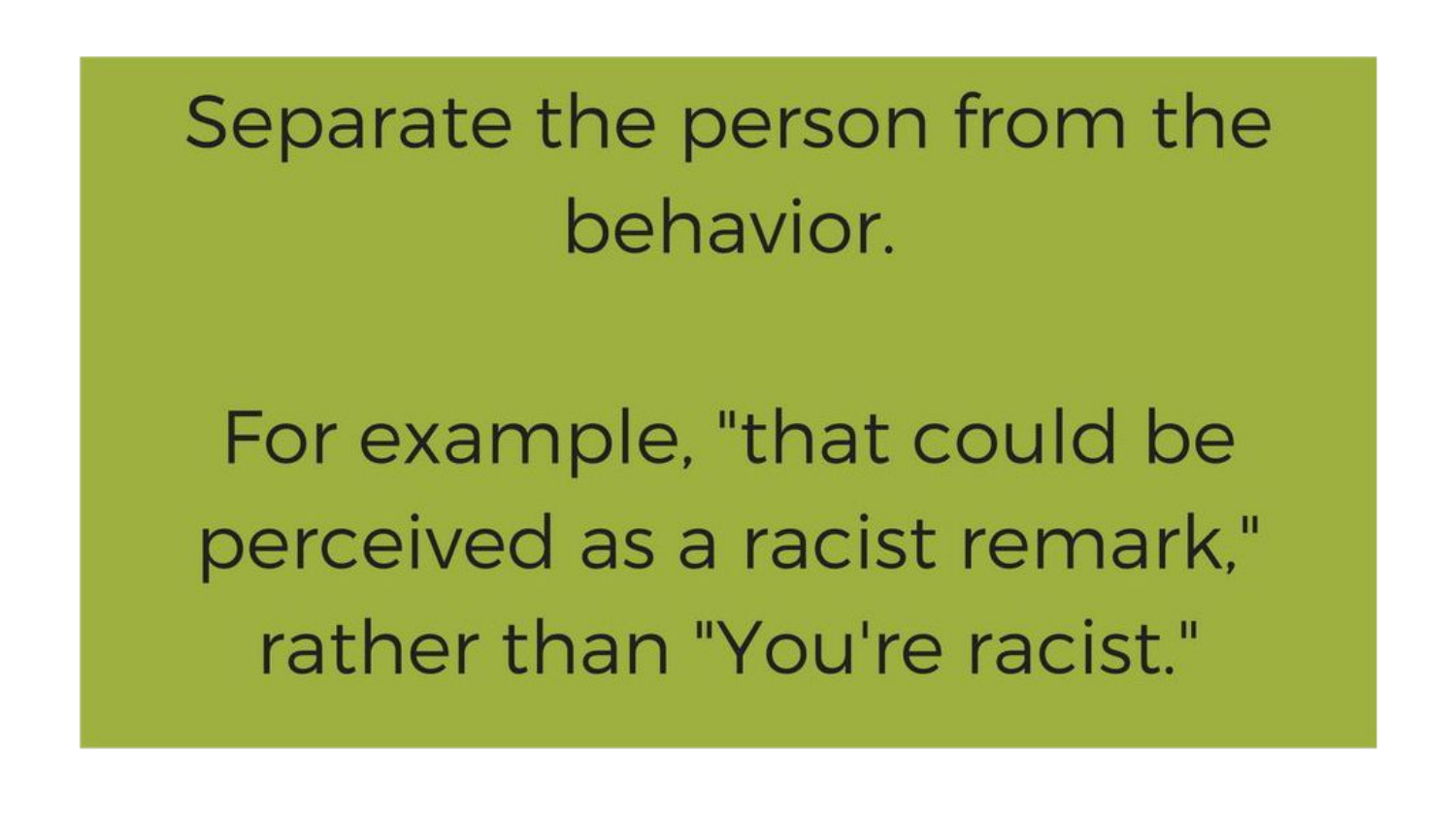

## Slide 16
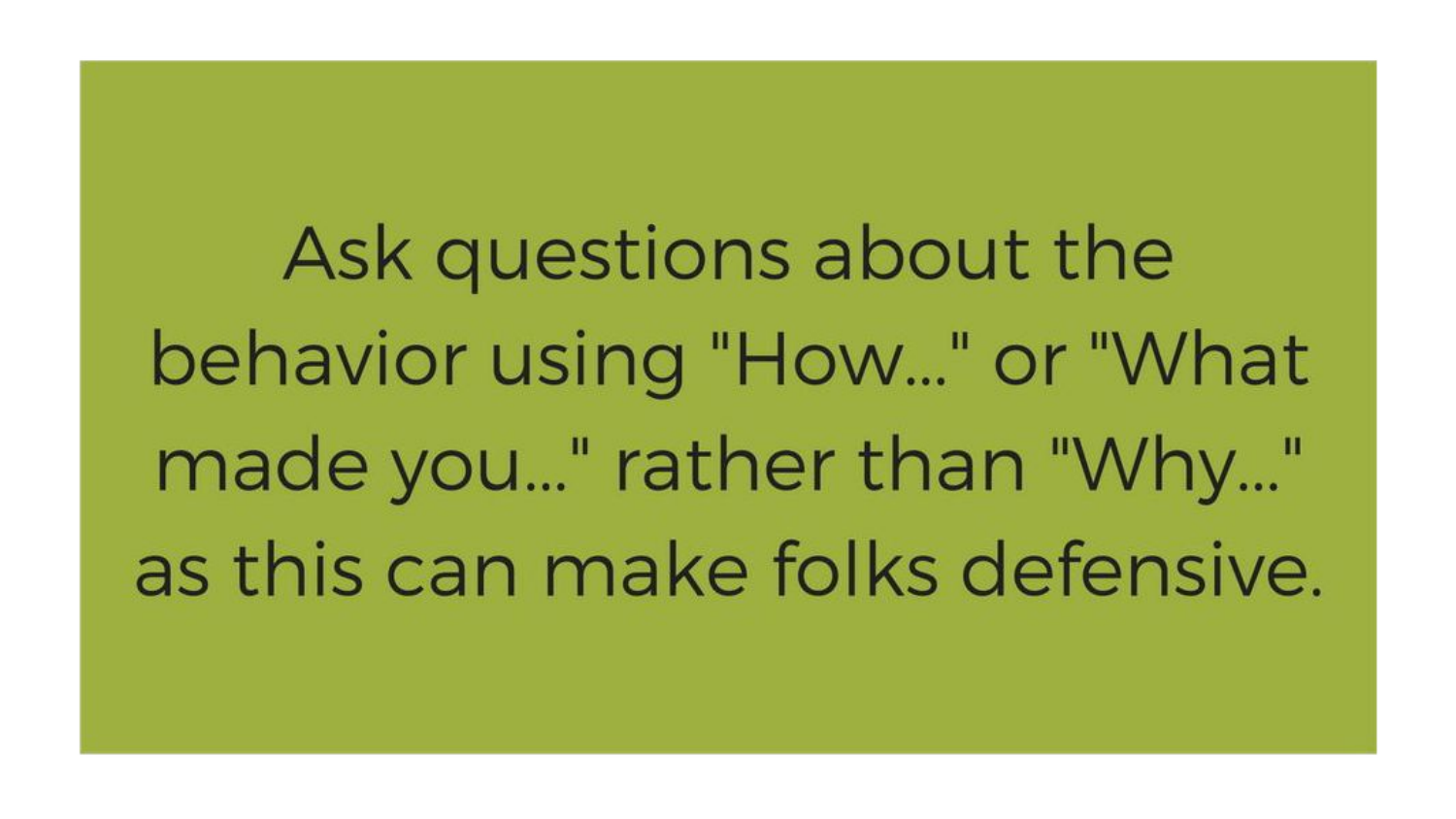

## Slide 17
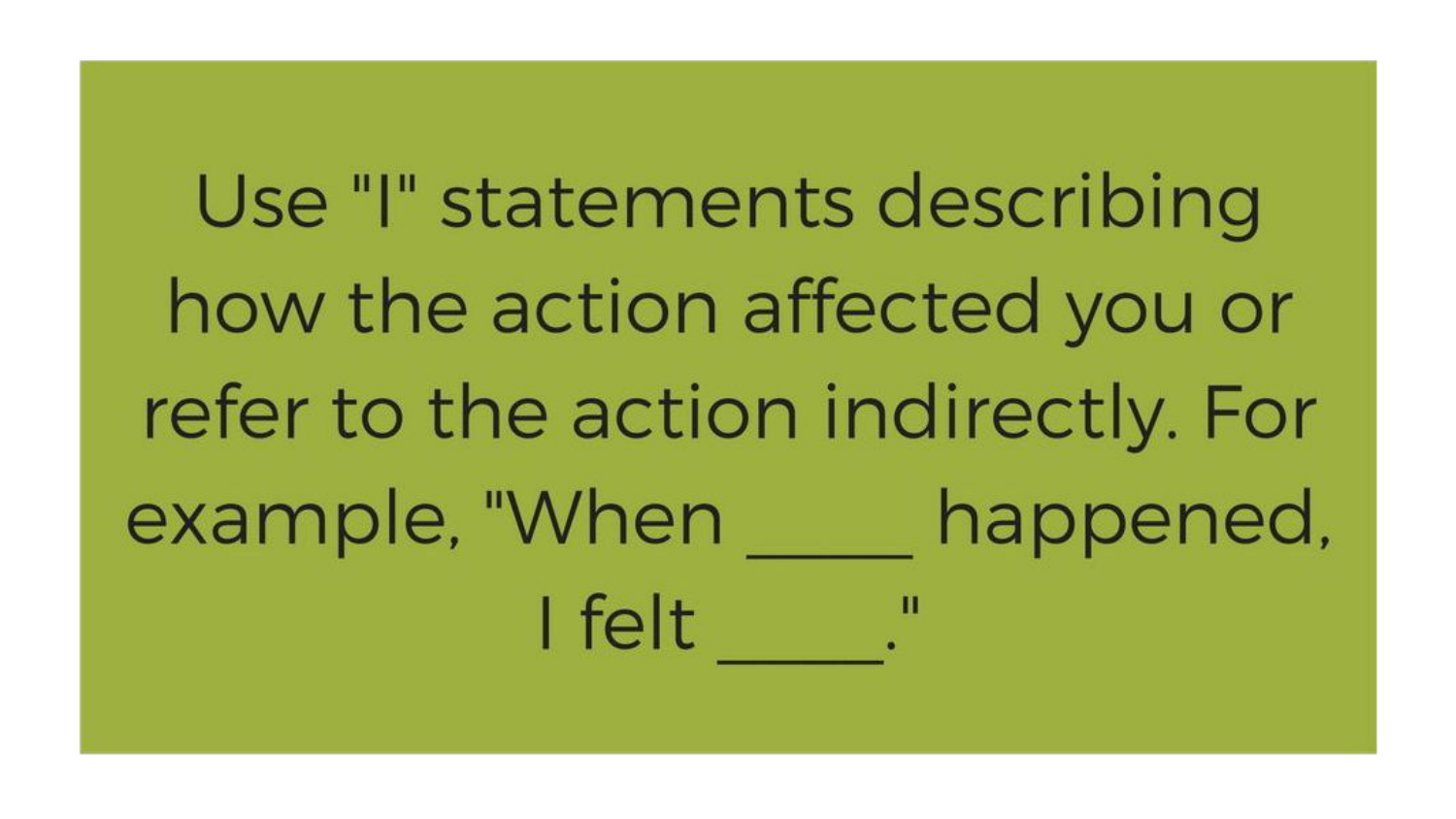

## Slide 18
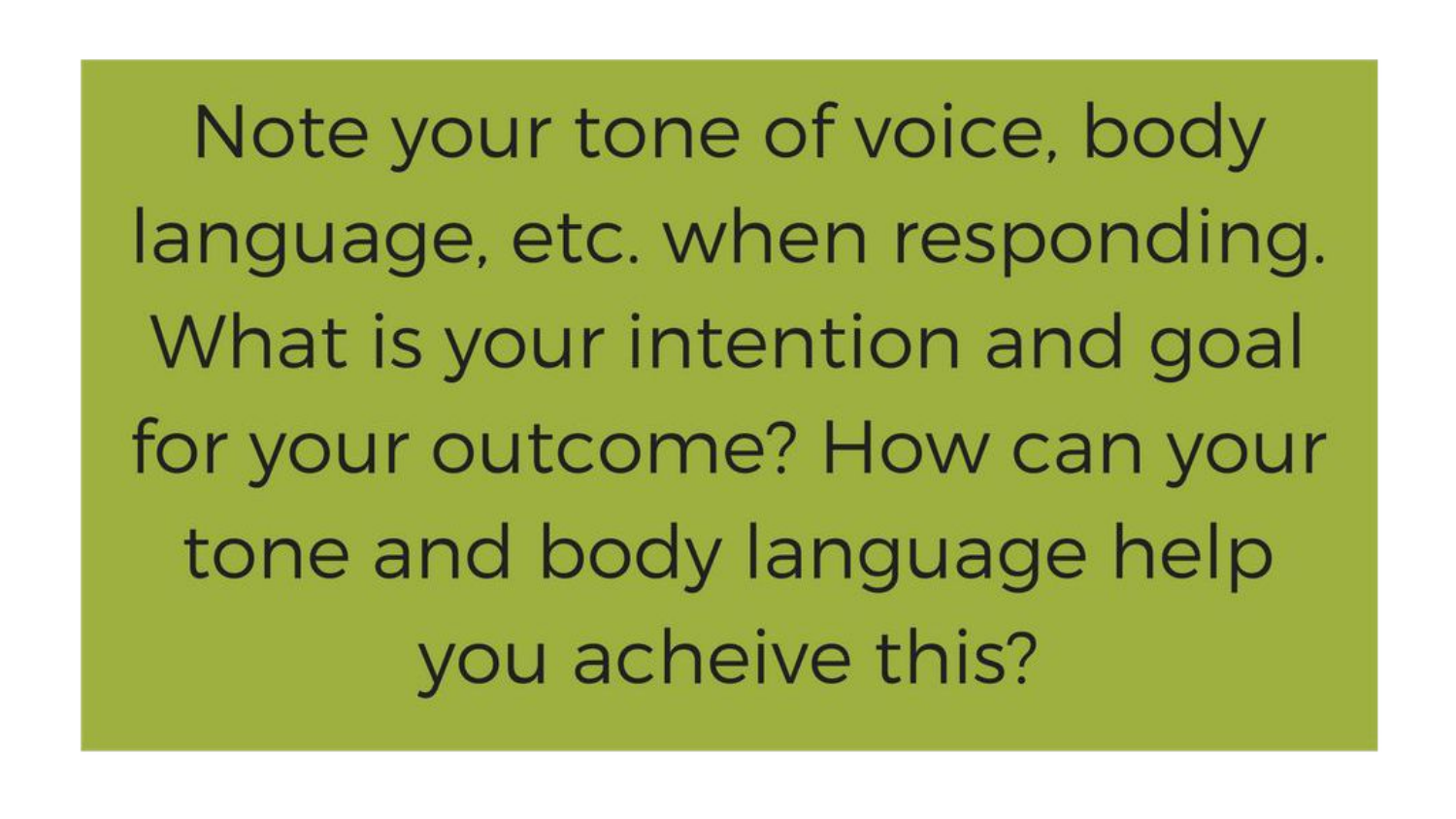

## Slide 19
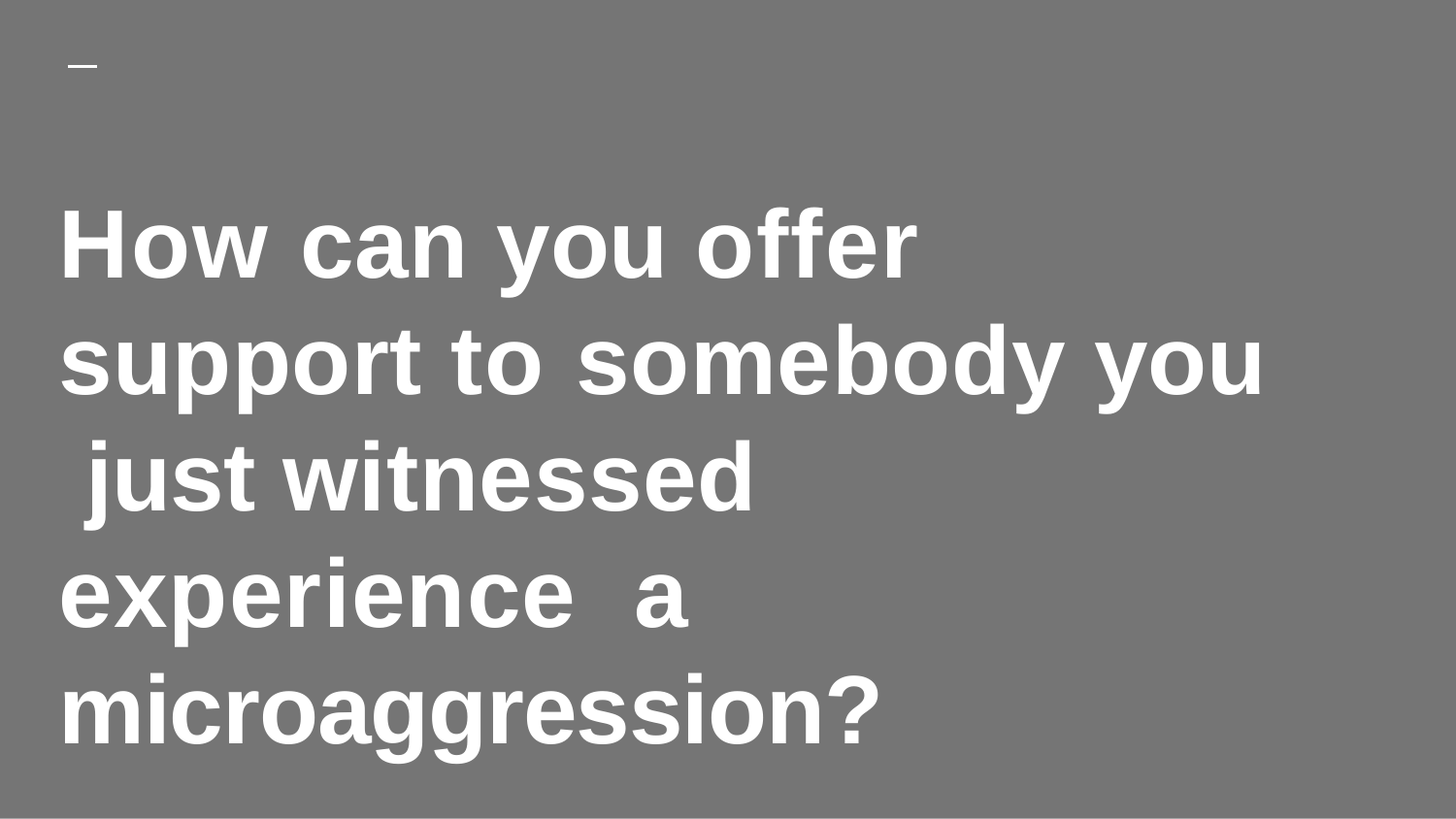

How can you offer support to somebody you just witnessed experience a microaggression?

## Slide 20
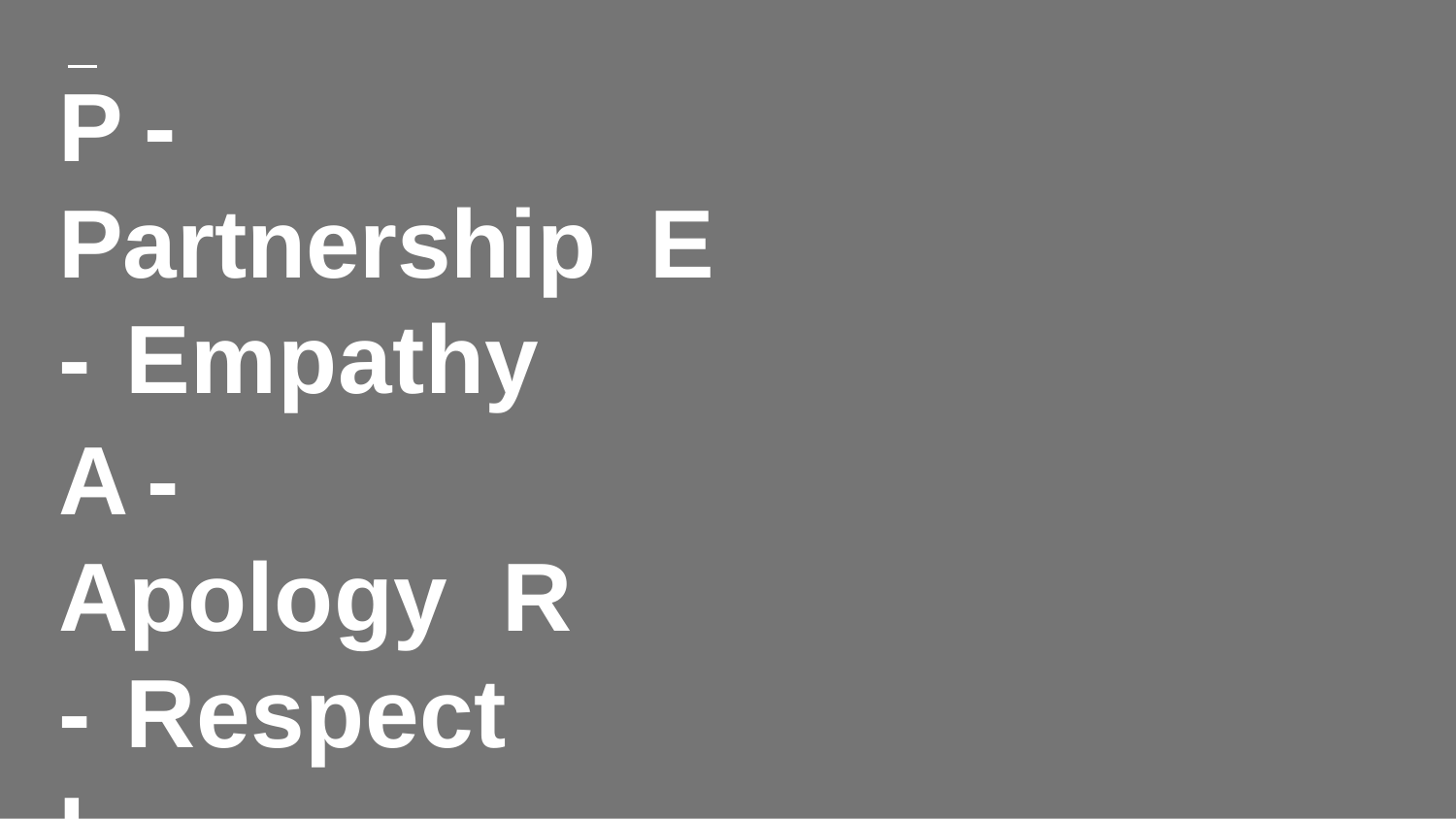

P - Partnership E - Empathy
A - Apology R - Respect
L - Legitimation S - Support

## Slide 21
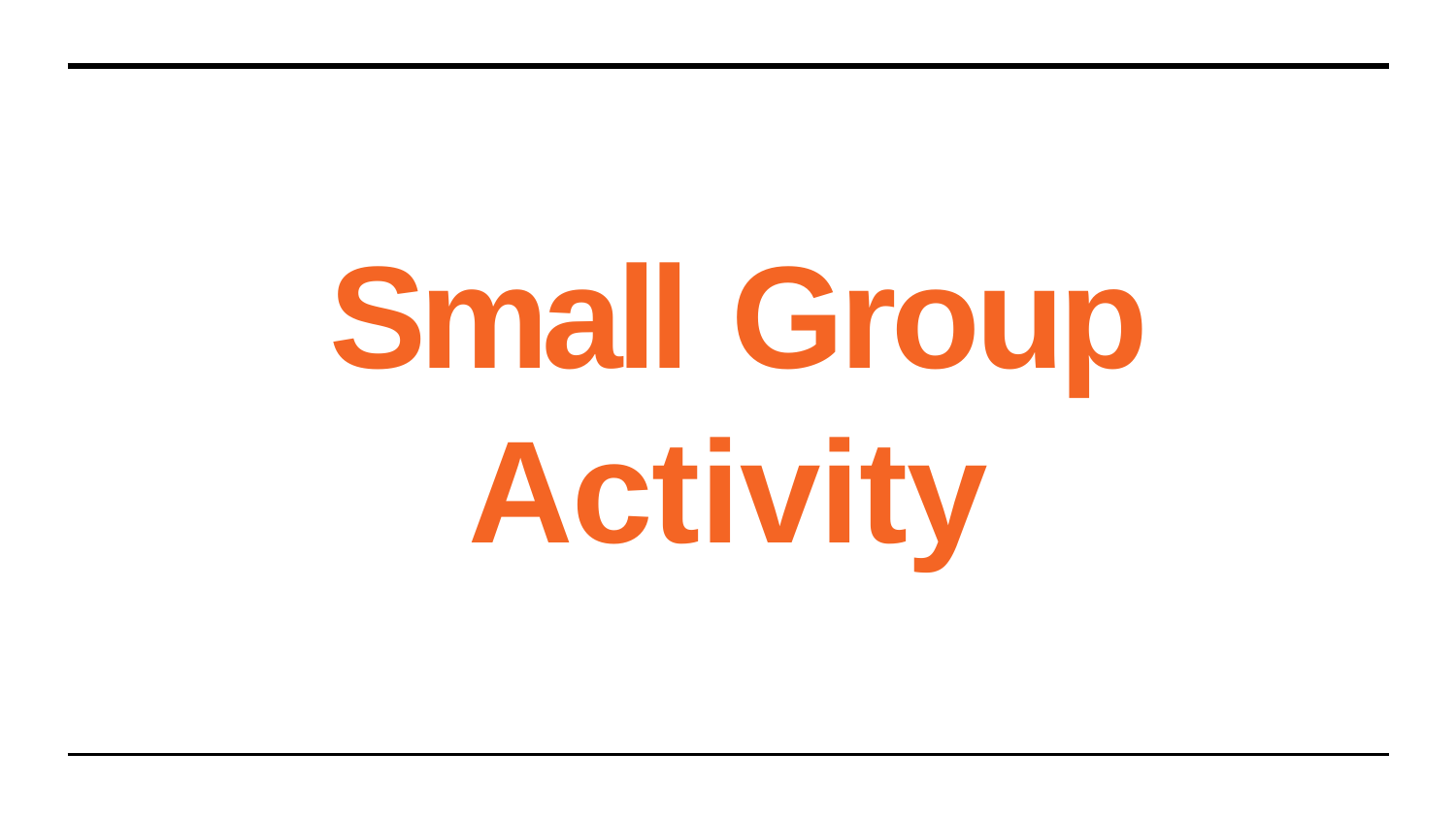

# Small Group Activity

## Slide 22
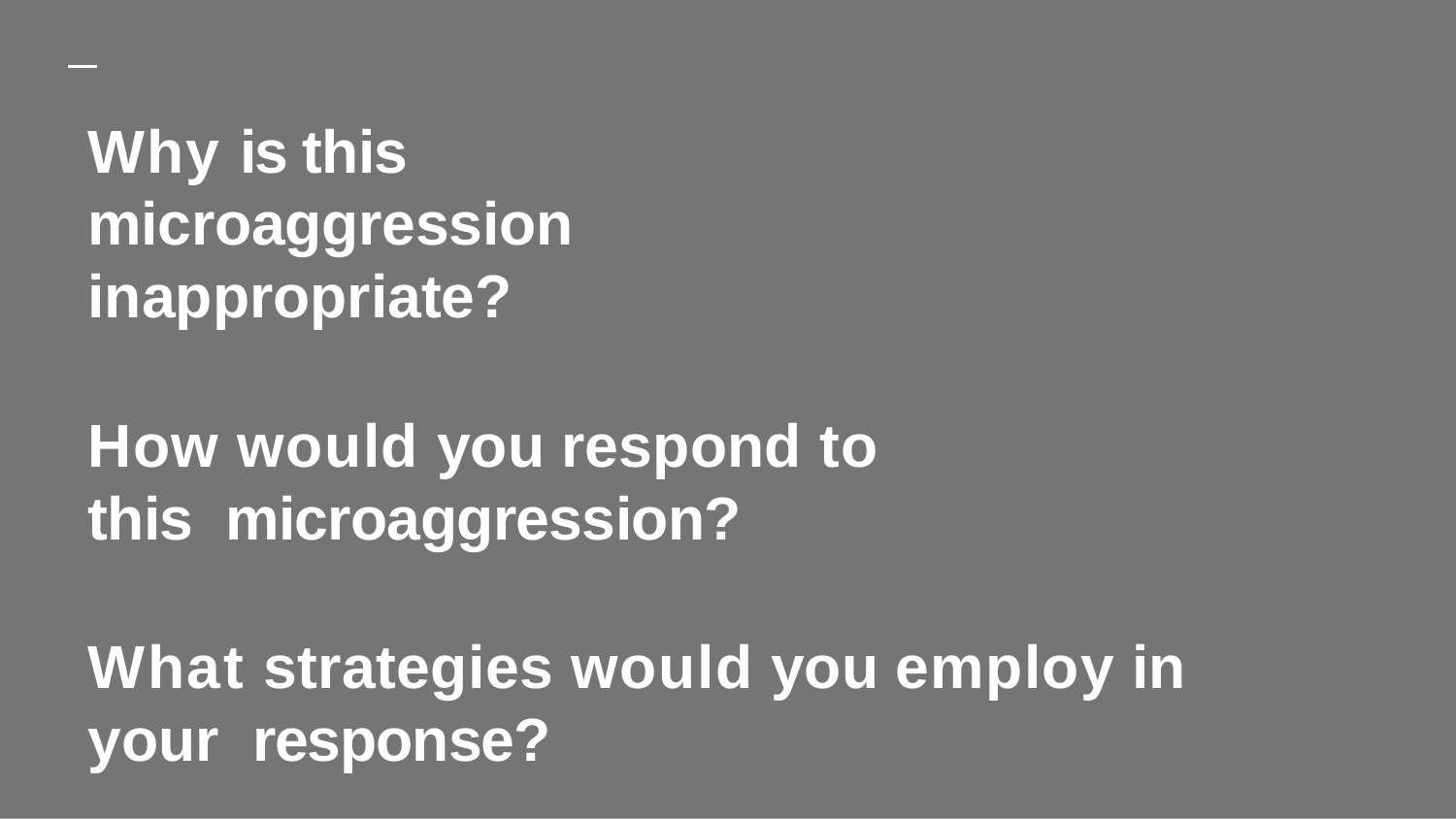

Why is this microaggression inappropriate?
How would you respond to this microaggression?
What strategies would you employ in your response?

## Slide 23
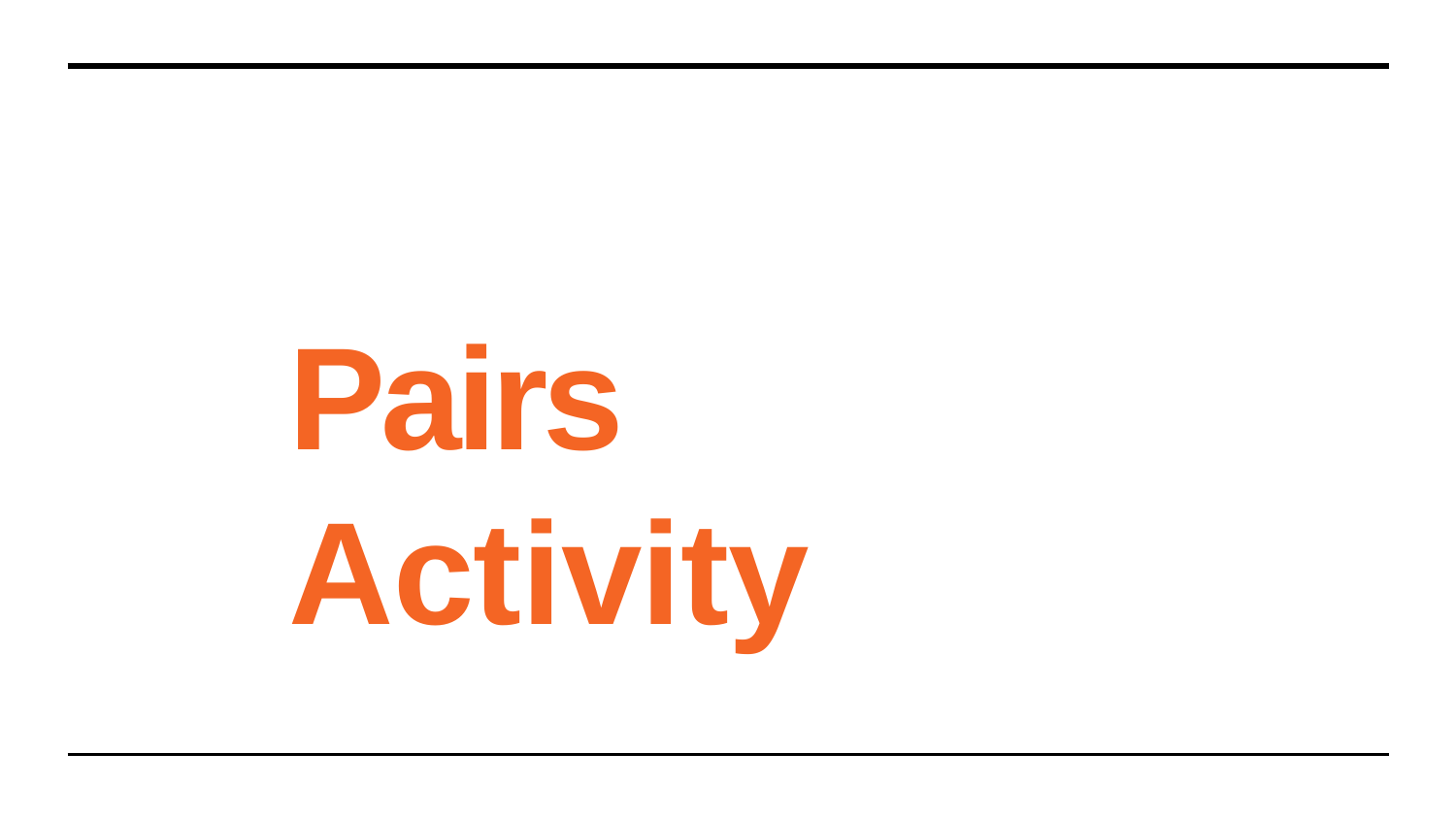

# Pairs Activity

## Slide 24
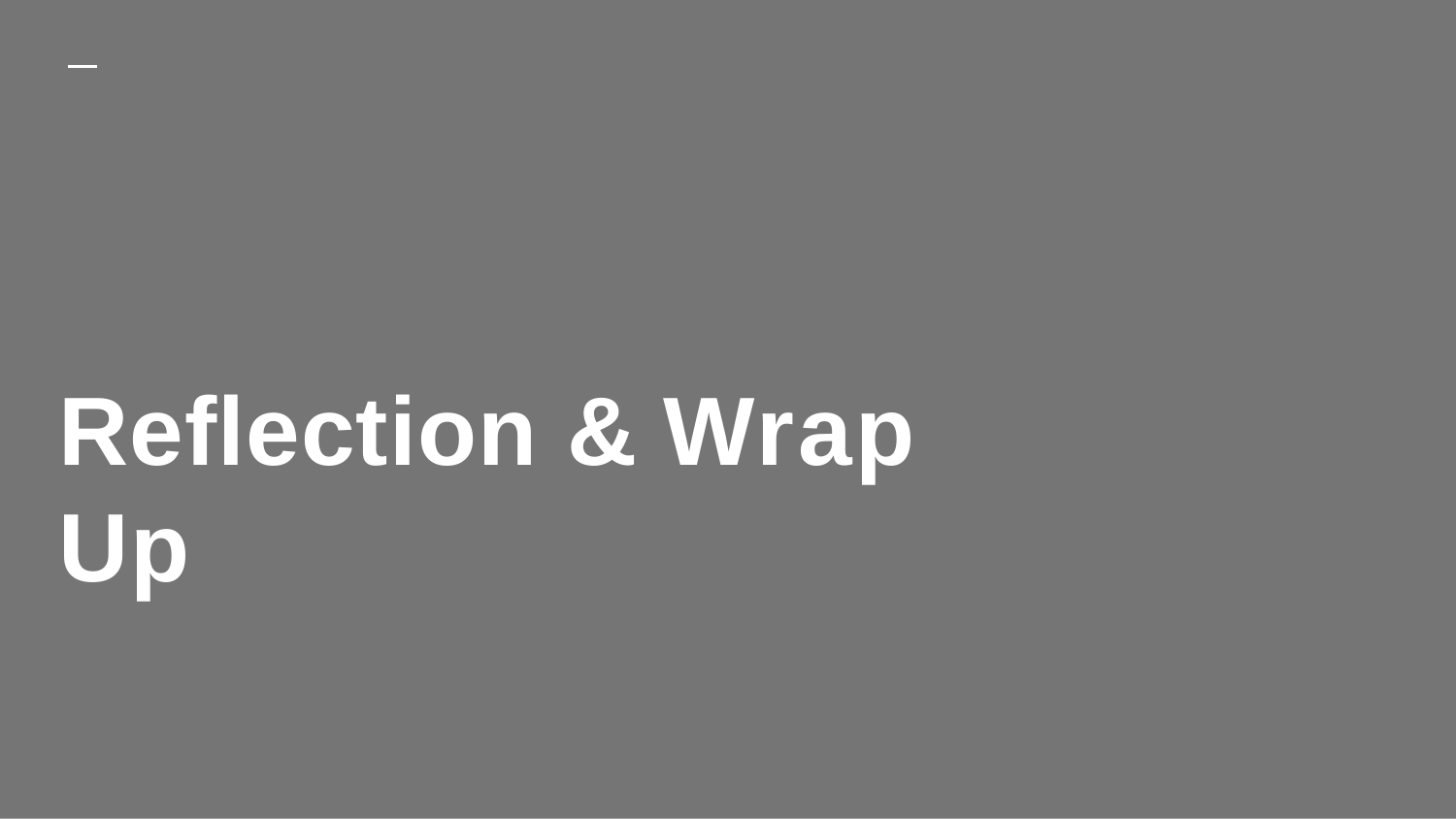

# Reflection & Wrap Up

## Slide 25
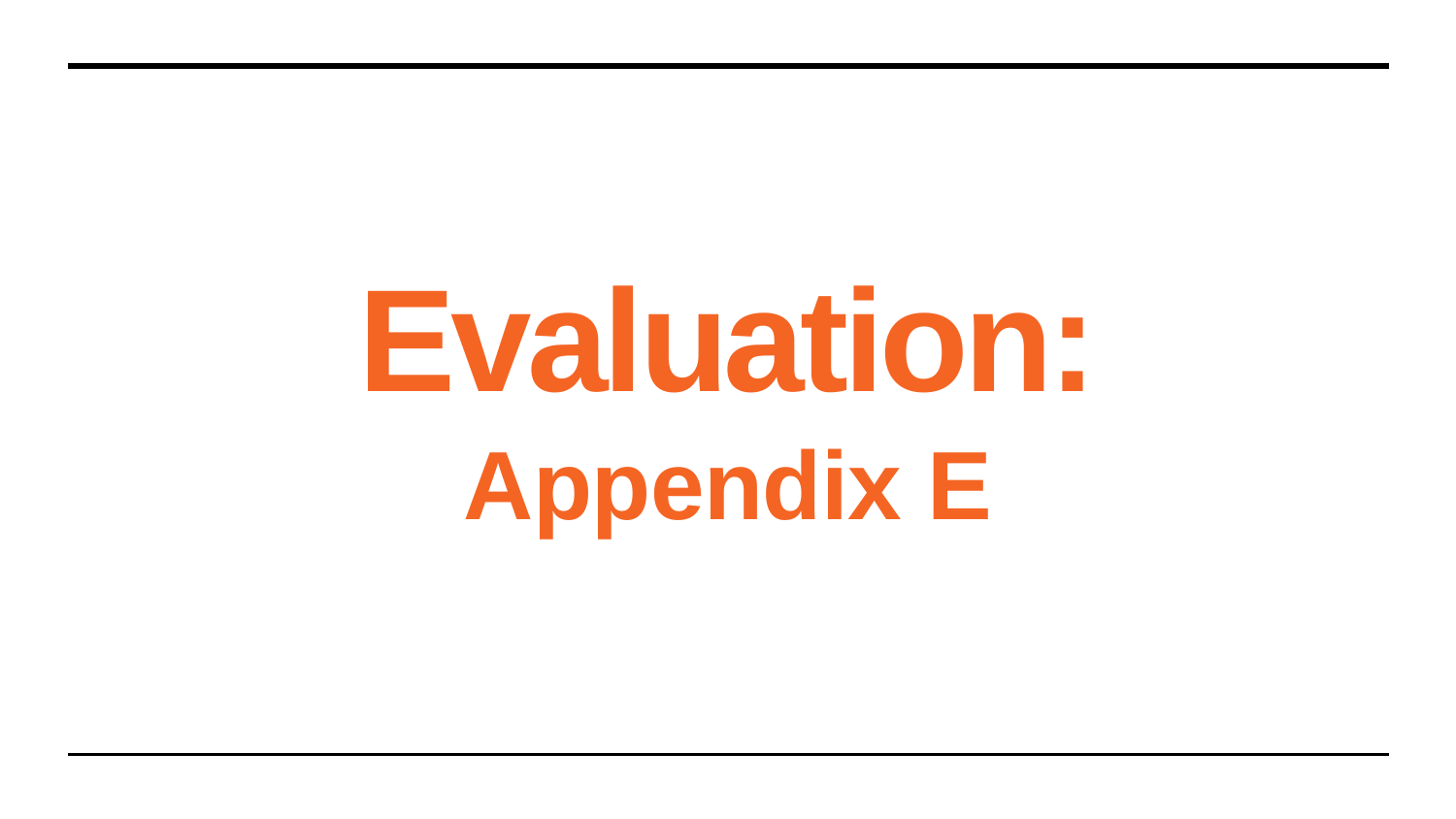

# Evaluation:
Appendix E
