# Supplementary material for: Interrupting Microaggressions in Health Care Settings: A Guide for Teaching Medical Students
Source: MedEdPORTAL. 2020 Jul 31;16:10969. doi: 10.15766/mep_2374-8265.10969 (PMC7394346; doi:10.15766/mep_2374-8265.10969)
Supplement: Supplementary file 1 — Preworkshop Survey.docxFacilitator Guide.docxWorkshop Presentation.pptxFaculty Development Agenda.docxPostworkshop Evaluation Form - Students.docxPostworkshop Debriefing Questions - Faculty.docx [file mep_2374-8265.10969-s001.zip › D. Faculty Development Agenda.docx]

**Interrupting Microaggressions in the Health Care Setting**

**Faculty Prep Session**

**AGENDA**

1. Introductions (5 minutes)
2. Background Information (10 minutes)
   1. Motivation/Rationale for discussion of microaggressions – Why is this topic important now?
   2. Personal Connection to Microaggressions – Please share your experiences.
3. Review of Facilitator Guide (see Appendix C) (35 minutes)
   1. Review of Roles in Workshop
   2. Emphasis of Importance of Group Agreements
   3. Review of Submitted Cases
      1. Review definitions of various -isms
      2. Discuss power dynamics in submitted cases
   4. Role Play Practice
4. Pitfall Planning (10 minutes)
   1. “Non-believer” vs. Feelings of personal attack
   2. “I submitted that case.”
   3. Others?
