# Supplementary material for: Interrupting Microaggressions in Health Care Settings: A Guide for Teaching Medical Students
Source: MedEdPORTAL. 2020 Jul 31;16:10969. doi: 10.15766/mep_2374-8265.10969 (PMC7394346; doi:10.15766/mep_2374-8265.10969)
Supplement: Supplementary file 1 — Preworkshop Survey.docxFacilitator Guide.docxWorkshop Presentation.pptxFaculty Development Agenda.docxPostworkshop Evaluation Form - Students.docxPostworkshop Debriefing Questions - Faculty.docx [file mep_2374-8265.10969-s001.zip › E. Postworkshop Evaluation Form - Students.docx]

**Interrupting Microaggressions in Health Care Settings: A Guide for Teaching Medical Students**

Post-Workshop Survey

Thank you so much for attending our workshop. Please fill out this short survey so we can improve this workshop in the future.

I feel confident…

|  | Strongly disagree | Disagree | Agree | Strongly agree |
| --- | --- | --- | --- | --- |
| Identifying microaggressions |  |  |  |  |
| Interrupting microaggressions when they occur |  |  |  |  |
| Supporting my peers and colleagues when they experience microaggressions |  |  |  |  |

The facilitators were….

|  | Strongly disagree | Disagree | Agree | Strongly agree |
| --- | --- | --- | --- | --- |
| Were well prepared |  |  |  |  |
| Created a welcoming and inclusive environment for discussion |  |  |  |  |
| Effectively communicated this information |  |  |  |  |

What did you like most about this workshop?

What did you like least about this workshop?

What other feedback or thoughts would you like to share?
