# Supplementary material for: Interrupting Microaggressions in Health Care Settings: A Guide for Teaching Medical Students
Source: MedEdPORTAL. 2020 Jul 31;16:10969. doi: 10.15766/mep_2374-8265.10969 (PMC7394346; doi:10.15766/mep_2374-8265.10969)
Supplement: Supplementary file 1 — Preworkshop Survey.docxFacilitator Guide.docxWorkshop Presentation.pptxFaculty Development Agenda.docxPostworkshop Evaluation Form - Students.docxPostworkshop Debriefing Questions - Faculty.docx [file mep_2374-8265.10969-s001.zip › F. Postworkshop Debriefing Questions - Faculty.docx]

**Interrupting Microaggressions in Health Care Settings: A Guide for Teaching Medical Students**

Facilitator Debriefing Questions

1. What went well?
2. What could be improved?
3. What challenges/pitfalls did you face?
